# Supplementary material for: The demographics and geographic distribution of laboratory-confirmed Lyme disease cases in England and Wales (2013–2016): an ecological study
Source: BMJ Open. 2019 Jul 31;9(7):e028064. doi: 10.1136/bmjopen-2018-028064 (PMC6677960; doi:10.1136/bmjopen-2018-028064)

Supplementary Material 1

The English Indices of Deprivation (EID) ranks 32,844 geographies (Lower super output areas) containing between 1000 and 3000 population and groups these in to deciles where 1 represents the areas with the highest levels of deprivation and 10 the lowest. The Welsh Index of Multiple Deprivation (WIMD) is ranked in a similar manner but is then grouped into the following categories; the 10% with the greatest deprivation, moving up through decreasing levels of deprivation in intervals from 10-20%, 20-30%, 30-50%, and the 50% least deprived areas. The EID and WIMD categorise each geographical area with a variety of deprivation domain scores to build a summary index figure (Supplementary Table 1).

Supplementary Table 1. Summary of the English Indices of Deprivation 2015[33] and Welsh Index of Multiple Deprivation[34] domains, and their weighting to calculate an Index of Multiple Deprivation

**English Indices of Deprivation (EID) Domain**	**Description**	**Weighting for construction of Index of Multiple Deprivation (IMD)**
Income Deprivation Domain	Proportion of population experiencing deprivation due to low income	22.5%
Employment Deprivation Domain	Proportion of working age population excluded from the labor market	22.5%
Education, Skills and Training Deprivation Domain	Measures the lack of attainment and skills in the local population	13.5%
Health Deprivation and Disability Domain	Measures the risk of premature death and the impairment of quality of life through poor physical or mental health	13.5%
Crime Domain	Measures the risk of personal and material victimisation at local level	9.3%
Barriers to Housing and Services Domain	Measures the physical and financial accessibility of housing and local services (schools, supermarkets, primary care and post offices)	9.3%
Living Environment Deprivation Domain	Measures the quality of the local environment (housing, air quality and road traffic accidents)	9.3%
**Index of Multiple Deprivation (IMD)**	**Overall measure of deprivation constructed by the weighted sum of the above domains**	
**Welsh Index of Multiple Deprivation (WIMD) Domain**	**Description**	**Weighting for construction of Welsh Index of Multiple Deprivation (WIMD)**
Income Domain	Proportion of population experiencing deprivation due to low income	23.5%
Employment Domain	Proportion of working age population excluded from the labor market	23.5%
Health Domain	Measures the lack of good health	14.0%
Education Domain	Measures the extent of deprivation relating to education, training and skills	14.0%
Access to Services Domain	Measures deprivation due to a house holds inability to access services considered necessary for day to day living.	10.0%
Community Safety Domain	Measures deprivation relating to living in a safe community	5.0%
Physical Environment Domain	Measures factors in the local area that may impact on wellbeing or quality of life	5.0%
Housing Domain	Measures deprivation through lack of adequate housing	5.0%
**Welsh Index of Multiple Deprivation (WIMD)**	**Overall measure of deprivation constructed by the weighted sum of the above domains**	

Supplementary Material 2

Supplementary Figure - Relationship between laboratory-confirmed Lyme disease case numbers (2013-2016) in England and the component measures of the English Indices of Deprivation 2015.

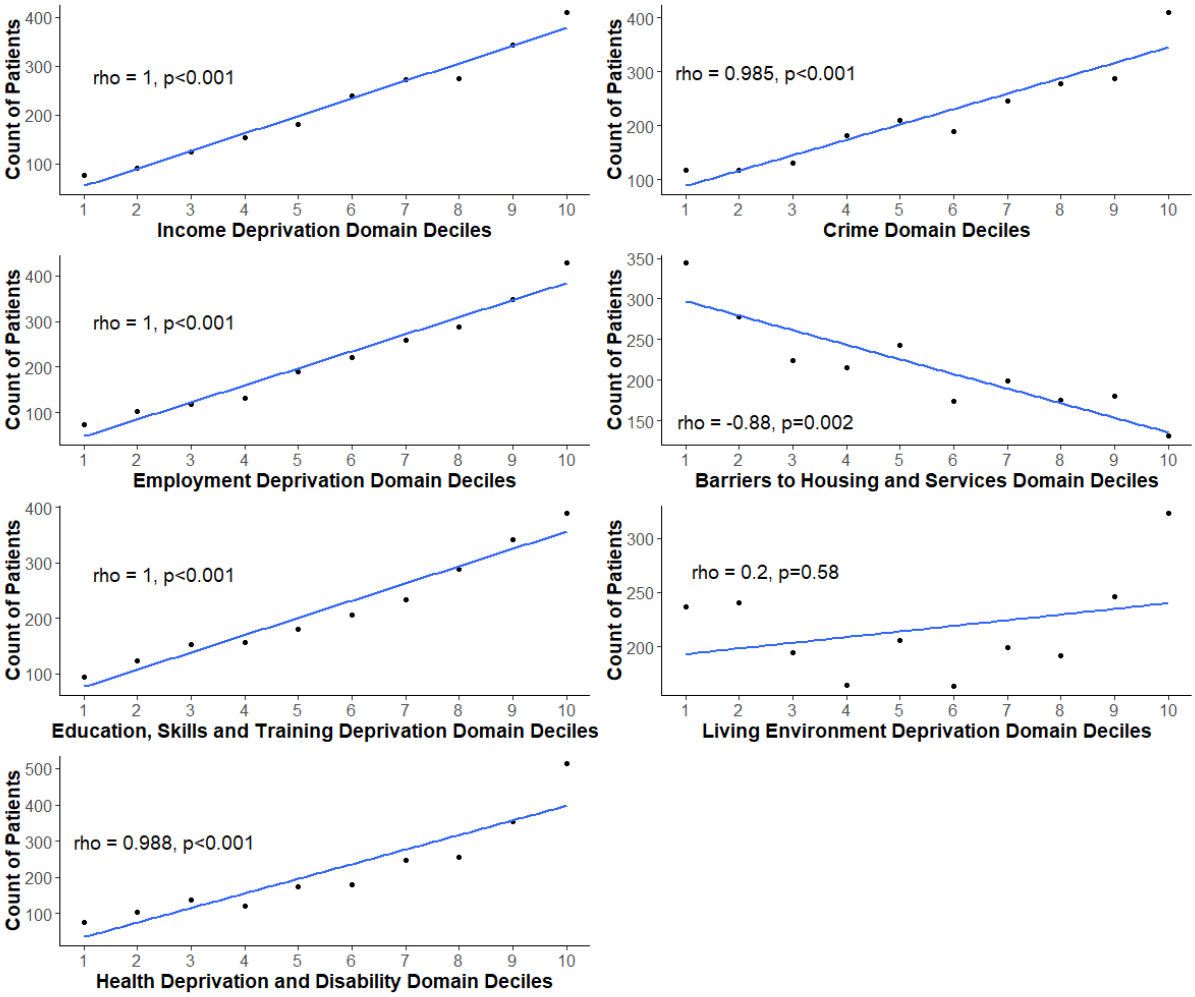

Supplement: Supplementary data [file bmjopen-2018-028064supp001.pdf]
