# Supplementary material for: The demographics and geographic distribution of laboratory-confirmed Lyme disease cases in England and Wales (2013–2016): an ecological study
Source: BMJ Open. 2019 Jul 31;9(7):e028064. doi: 10.1136/bmjopen-2018-028064 (PMC6677960; doi:10.1136/bmjopen-2018-028064)
Supplement: Author's manuscript [file bmjopen-2018-028064.draft_revisions.pdf]

BMJ Open is committed to open peer review. As part of this commitment we make the peer review history of every article we publish publicly available.

When an article is published we post the peer reviewers' comments and the authors' responses online. We also post the versions of the paper that were used during peer review. These are the versions that the peer review comments apply to.

The versions of the paper that follow are the versions that were submitted during the peer review process. They are not the versions of record or the final published versions. They should not be cited or distributed as the published version of this manuscript.

BMJ Open is an open access journal and the full, final, typeset and author-corrected version of record of the manuscript is available on our site with no access controls, subscription charges or pay-per-view fees (http://bmjopen.bmj.com).

If you have any questions on BMJ Open's open peer review process please email info.bmjopen@bmj.com

BMJ Open

The demographics and geographic distribution of laboratory-confirmed Lyme disease cases in England and Wales; 2013-2016

Journal:	*BMJ Open*
Manuscript ID	bmjopen-2018-028064
Article Type:	Research
Date Submitted by the Author:	20-Nov-2018

Complete List of Authors:	Tulloch, John; University of Liverpool, NIHR Health Protection Research Unit in Emerging and Zoonotic Infections; Public Health England<br>Semper, Amanda; Public Health England, NIHR Health Protection Research Unit in Emerging and Zoonotic Infections; Public Health England, Rare and Imported Pathogens Laboratory<br>Brooks, Tim; Public Health England, NIHR Health Protection Research Unit in Emerging and Zoonotic Infections; Public Health England, Rare and Imported Pathogens Laboratory<br>Russell, Katherine; Public Health England<br>Halsby, Kate; Public Health England<br>Christley, Robert; University of Liverpool, NIHR Health Protection Research Unit in Emerging and Zoonotic Infections; University of Liverpool, Institute of Infection and Global Health<br>Radford, Alan; University of Liverpool, NIHR Health Protection Research Unit in Emerging and Zoonotic Infections; University of Liverpool, Institute of Infection and Global Health<br>Vivancos, Roberto; Public Health England, Field Epidemiology Services; Public Health England, NIHR Health Protection Research Unit in Emerging and Zoonotic Infections<br>Warner, Jenny; Public Health England, NIHR Health Protection Research Unit in Emerging and Zoonotic Infections; Public Health England, Rare and Imported Pathogens Laboratory

Keywords:	Lyme disease, Lyme borreliosis, Epidemiology < INFECTIOUS DISEASES, England, surveillance, laboratory

SCHOLARONE™
Manuscripts

Title: The demographics and geographic distribution of laboratory-confirmed Lyme disease cases in England and Wales; 2013-2016

Authors: John S.P. Tulloch[1,2], Amanda E. Semper[3,4], Tim J.G. Brooks[3,4], Katherine Russell[5], Kate D. Halsby[5], Rob M. Christley[1,6], Alan D. Radford[1,6], Roberto Vivancos[2,7], Jenny C. Warner[3,4]

1. NIHR Health Protection Research Unit in Emerging and Zoonotic Infections, University of Liverpool, L69 3GL, UK.
2. Public Health England, Liverpool, L3 1DS, UK
3. NIHR Health Protection Research Unit in Emerging and Zoonotic Infections, Public Health England, Porton Down, SP4 0JQ, UK.
4. Rare and Imported Pathogens Laboratory, Public Health England, Porton Down, SP4 0JQ, UK
5. Public Health England, London, NW9 5EQ, UK
6. Institute of Infection and Global Health, University of Liverpool, Liverpool, CH64 7TE, UK
7. NIHR Health Protection Research Unit in Emerging and Zoonotic Infections, Public Health England, Liverpool, L3 1DS, UK.

Corresponding authors: John Tulloch; jtulloch@liverpool.ac.uk; Public Health England, Cunard Building, Water Street, Liverpool, L3 1DS, UK; +44 0344 225 0562

Jenny Warner; jenny.warner@phe.gov.uk; Rare and Imported Pathogens Laboratory, Public Health England, Porton Down, SP4 0JQ, UK; +44 01980 612899

Word count: 3571

For peer review only - http://bmjopen.bmj.com/site/about/guidelines.xhtml

For peer review only

Title: The demographics and geographic distribution of laboratory-confirmed Lyme disease cases in England and Wales; 2013-2016

Abstract

Objective: Lyme disease is a tick-borne disease of increasing incidence and public concern across the Northern Hemisphere. However, the socio-demographics and geographic distribution of the population affected in England and Wales are poorly understood. Therefore, the proposed study was designed to describe the demographics and distribution of laboratory-confirmed cases of Lyme disease from a national testing laboratory.

Design: An ecological study of routinely collected laboratory surveillance data

Setting: Public Health England's national Lyme disease testing laboratory.

Participants: 3,986 laboratory-confirmed cases of Lyme disease between 2013 and 2016.

Results: In England and Wales, the incidence of laboratory-confirmed Lyme disease rose significantly over the study period, from 1.62 cases per 100,000 in 2013 to 1.95 cases per 100,000 in 2016. There was a bimodal age distribution (with peaks at 6-10 and 61–65 years age bands) with a predominance of male patients. A significant clustering of areas with high Lyme disease incidence was located in southern England. An association was found between disease incidence and socioeconomic status, based on the patient's resident postcode, with more cases found in less deprived areas. Cases were disproportionately found in rural areas compared to the national population distribution.

Conclusions: These results suggest that Lyme disease patients originate from areas with higher socioeconomic status and disproportionately in rural areas. Identification of the Lyme

For peer review only - http://bmjopen.bmj.com/site/about/guidelines.xhtml

disease hotspots in southern England, alongside the socio-demographics described, will enable a targeted approach to public health interventions and messages.

Keywords: Lyme disease; Lyme borreliosis; epidemiology; England; Wales; surveillance; laboratory

Strengths and limitations of this study

- This study is based upon a national testing laboratory's figures and provides a much needed update on basic epidemiological information about Lyme disease in England and Wales.
- Data on the socio-economic status of Lyme disease cases is globally sparse; our findings will have implications for future public health awareness and intervention schemes and may offer new avenues for research.
- Lyme disease incidence maps have been produced to a high resolution and show significant clustering of disease; providing public health organisations with locales to target interventions.
- Geographical data, and associated variables, were based upon patient residence information rather than tick bite location.
- The study was of an ecological design and positive cases were compared to the national population, therefore no measures of risk or multivariable analysis of demographic variables were possible.

For peer review only

Introduction

Lyme disease is an important zoonotic tick-borne disease caused by spirochaetes of the *Borrelia burgdorferi sensu lato* genospecies complex. It is spread through the bites of infected *Ixodes* ticks, in the United Kingdom (UK) primarily *Ixodes Ricinus.*[1] Autochthonous cases are found solely in the northern hemisphere.[2,3] Most commonly, early infection presents with an erythema migrans rash, with associated generalised flu-like symptoms.[4] Neurological manifestations, such as facial nerve palsy, can occur as part of early disseminated infection.[2] The varied presentation of the disease and the potential of increased tick exposure risk due to the extension of tick habitats as a result of changes in land management, climate and human activity, has resulted in heightened awareness and surveillance by public health organisations.[5,6]

In Western Europe the population-weighted incidence has been estimated at 22.04 cases per 100,000 person-years.[7] In the UK, Lyme disease is not a notifiable disease, but laboratory-confirmed *Borrelia* spp. are notifiable causative organisms.[8] Public Health England (PHE) complies data on laboratory-confirmed cases of Lyme disease in England and Wales, which show a rise in the national incidence of confirmed cases from 0.38 per 100,000 population in 1997[9] to 1.95 per 100,000 population in 2016.[10] Data on laboratory-confirmed cases are provided by the national diagnostic laboratory, the PHE Rare and Imported Pathogens Laboratory (RIPL), which provides specialist advice and diagnostics for Lyme disease to the National Health Service (NHS) in England and Wales. Laboratory testing is based on serological diagnosis using a combination of screening and confirmatory immunoassays in accordance with internationally accepted best practice for Lyme disease diagnosis.[4,11,12] The incidence of cases which do not require laboratory diagnostics is unknown. These cases are most likely

For peer review only

For peer review only - http://bmjopen.bmj.com/site/about/guidelines.xhtml

presented to and are clinically-diagnosed and managed solely within primary care, as recommended by The National Institute for Health and Care Excellence (NICE) guidelines.[4]

Information regarding the demographics of Lyme disease cases in England and Wales is limited. Laboratory surveillance data published in 2000 describe an equal sex ratio at all ages, however, numbers were not provided and statistical comparison was not performed.[13] They describe a bimodal age distribution with peaks in childhood and at 45-64 years old. Hospital admissions data investigating Lyme disease and Bell's palsy describe a similar bimodal distribution.[14] These findings are similar to other European countries.[15–17] There is a sparsity of recent demographic data for Lyme disease in England and Wales. The geographic distribution of confirmed cases was last described in 2000.[13] They describe a tendency for cases in southern England, especially around the New Forest. However, this data may not reflect the current distribution of Lyme disease cases in England and Wales. More current data is urgently needed to enable targeted public health messaging and intervention strategies.

Globally, the negative income and education gradient of health has helped shape public health strategy and policy.[18,19] As a person's position on the socioeconomic spectrum increases, so their likelihood of better health increases. Such potentially avoidable disparities in health has led to an increased focus on understanding the social determinants of health[20] and developing measures to address these. Work to explore the association between socioeconomic status and Lyme disease incidence is limited. In the United States of America (USA) persons were to found to be at greatest risk of Lyme disease if they lived in the highest or lowest socially vulnerable areas.[21] Two studies found a relationship between Lyme disease incidence and median annual household income, with incidence peaking at around

For peer review only - http://bmjopen.bmj.com/site/about/guidelines.xhtml

80,000 USD.[22,23] However, a consistent relationship between the socioeconomic state of an individual and their Lyme disease acquisition risk has yet to emerge. In particular, no in-depth research has been performed in Europe investigating the socioeconomics of the Lyme disease patient cohort.

The aim of this study was to utilise information collected through routine surveillance in England and Wales to describe the demographics and geographic distribution of laboratory-confirmed Lyme disease cases over a four year period. Correlations between Lyme disease incidence and socioeconomic indices were analysed, using patient residence postcode as a proxy for individual patient characteristics. New insight will be provided into the key demographic, geographical and social determinants of the Lyme disease patient population. This would allow us to identify potentially at-risk populations, shape public health interventions and assist in appropriate disease awareness.

For peer review only

Method

A retrospective analysis was performed using data extracted from the PHE Rare and Imported Pathogens Laboratory's (RIPL) laboratory information management system (LIMS), between 1st January 2013 and 31st December 2016, for laboratory-confirmed Lyme disease cases, the same data as used for PHE's Zoonoses Report.[24] The RIPL LIMS contains information provided on the Lyme disease referral form submitted at the time of sample submission and any additional information provided by clinicians during case follow up and management.[25] The form captures information on the age, gender, location, clinical symptoms and travel history of the patient. Data were cleaned and duplicates were removed where necessary.

Annual Lyme disease incidence estimates were calculated, using the Office for National Statistics (ONS) mid-year population estimates as the denominator population.[26] A Chi-

For peer review only - http://bmjopen.bmj.com/site/about/guidelines.xhtml

squared test for trend and a Chi-squared test for departure from the trend were used to analyse trends in incidence. Cases were stratified by age and gender. Using binomial tests, the null hypothesis that there was no difference in case numbers between males and females was tested within differing age bands, and overall.

Geographical information was collated based on (1) the regional origin of a diagnostic sample (usually a hospital microbiology department) consisting of eight PHE regions, and Wales as a whole,[27] and (2) the postcode area of the patient. These were used to calculate average annual incidence for the study period. In an attempt to account for the unknown distance between a patient's home address and where they were bitten, the disease incidence map for postcode area was smoothed using a k-nearest neighbours (k-NN) approach;[28–30] this was In this approach, k is defined as the number of neighbours used for smoothing and is equal to the square root of the total number of discrete geographical areas rounded to the nearest whole odd number (i.e. 105 postcode areas, its square root being 10.2, therefore k=11). Exploratory spatial data analysis (EDSA)[31,32] was used to explore the spatial autocorrelation of the postcode area incidence map. Global and local Moran's I values were calculated, and a LISA (Local Indicators of Spatial Association) significance map constructed to highlight any significant clusters. In both the k-NN smoothing and Moran's I calculations, a queen adjacency matrix was used.

Patient postcode was linked to ONS socioeconomic data,[26] enabling a description of the socioeconomic characteristics of the population in which a Lyme disease case was resident. If no patient postcode was recorded, these cases were excluded from the analysis. Socioeconomic status is reported through the English Indices of Deprivation (EID) 2015[33] and the Welsh Index of Multiple Deprivation (WIMD) 2014[34] (Supplementary material 1).

For peer review only - http://bmjopen.bmj.com/site/about/guidelines.xhtml

Postcode area case count data were matched independently to the EID and WIMD, and a rural urban classification. As EID and WIMD are on a discrete ordinal scale, Spearman's rank correlation was used to calculate the correlation between the number of cases and deprivation score. The proportion of cases with their home addresses located in either a rural or urban area, were compared to the national rural urban classification from the ONS.[35] This was performed using a Chi-squared test of independence for both English and Welsh data.

All statistical and spatial analyses were carried out using R language (version 3.2.0) (R Core Team 2015). Results were deemed significant where $p<0.05$.

Results

In total 3,986 unique cases, 3,893 cases in England and 93 in Wales, meeting a serological diagnosis of Lyme disease were identified in the RIPL LIMS between 1st January 2013 and the 31st December 2016. Of these, 98.7% (n=3,935) had complete records for date of submission, gender and age.

The annual incidence of laboratory-confirmed Lyme disease cases in England and Wales rose from 1.62 per 100,000 population in 2013, to 1.95 in 2016. These figures are identical to PHE's official incidence figures as they used the same data source.[10] There was evidence of an overall association between incidence and year (χ^2=43.13, $p<0.001$). This association took the form of a trend with increasing incidence each year (χ^2=30.17, $p<0.001$). Departures from the trend were significant (χ^2=43.1-30.1=12.96, $p<0.001$), as shown by the fall in incidence in 2014. There was marked seasonality, with the peak numbers of cases being diagnosed in the summer months each year (Fig 1).

For peer review only - http://bmjopen.bmj.com/site/about/guidelines.xhtml

Across all ages there were significantly more male (n=2,096) than female (n=1,839) cases (p < 0.001), with a bimodal age distribution, with peaks at 6-10 and 61-65 year age bands (Fig 2). Grouping the data in 5-year age bands, there were significantly more men than women in the 6-10 (p=0.03), 11-15 (p=0.03), 36-40 (p=0.01), 41-45 (p=0.02), and 46-50 (p=0.04) age groups.

Data were available about PHE regions for 99.9% (n=3,985) of the study population (Fig 3a), and about patient residence postcode area for 58.2% (n=2,321). The South West PHE region had the highest incidence of Lyme disease in England and Wales; none of the PHE regions, nor Wales, reported zero cases. The postcode areas with the highest average annual incidence of Lyme disease were Southampton (11.65 cases per 100,000 per year), Salisbury (10.75), Bournemouth (5.62), Reading (4.59), Dorchester (4.57), Guildford (4.31), Taunton (2.79), Torquay (2.75), Brighton (1.96), and Bath (1.84) (Fig 3b). These areas are all in southern England. Only four postcode areas had no laboratory-confirmed cases in the four year surveillance period (Fig 3b), namely Dartford, Eastern Central London, Hull, and Western Central London. The smoothed data showed a trend for the areas of highest incidence to be located in southern-central England (Fig 3c). There was significant spatial autocorrelation, the global Moran's I was 0.564 (p=0.01), indicating that postcode areas with similar incidence are clustered together. LISA mapping identified six areas as significant clusters of high incidence (Fig 3d); Southampton, Salisbury, Bournemouth, Reading, Dorchester, and Guildford (for all p<0.001).

Using patient residence postcode data, it was possible to match 55.6% (n=2,165) of English records to the English Indices of Deprivation and 98.2% (n=92) of Welsh records to the Welsh Index of Multiple Deprivation (WIMD). An overall significant positive correlation between the number of cases and Index of Multiple Deprivation (IMD) decile was observed (ρ=0.96,

For peer review only - http://bmjopen.bmj.com/site/about/guidelines.xhtml

p<0.001), with more Lyme disease cases found in less deprived areas (Fig 4). This significant positive correlation was seen across all domains of deprivation, except the 'Barriers to Housing and Services Domain' where this trend was reversed (ρ=-0.88, p=0.002) and the 'Living Environment Deprivation Domain' where there was no significant correlation (ρ=0.2, p=0.58) (Supplementary material 2). An overall significant positive correlation between the number of cases and WIMD rank was observed (ρ=0.89, p=0.04), with more Lyme disease cases found in the least deprived areas.

When compared to the national population, the study population was disproportionately more likely to live in a rural area, for both English (p<0.001) and Welsh (p<0.001) sections of the study population (Table 1).

Table 1 – The rural urban classification of laboratory-confirmed cases of Lyme disease in England and Wales (2013-2016) compared to the national census population

Category	Percentage of English Study Population	Percentage of Welsh Study Population	Percentage of 2015 census population
Rural	34.3% (n=743)	47.8% (n=44)	17.9%
Urban	65.7% (n=1,422)	52.2% (n=48)	82.1%

Discussion

Between 2013 and 2016 there was a significant increase in annual incidence of cases of confirmed Lyme disease, with a seasonality that matched previous publications and has been well documented.[9] The observed seasonality closely matches *I. ricinus* tick population dynamics in the UK, which annually peak around June and July.[1,36] Concerns have been raised about how the expansion of tick habitats due to changes in land use and management, and climate change, may be increasing the risk of Lyme disease infection.[5,37] Although the

For peer review only - http://bmjopen.bmj.com/site/about/guidelines.xhtml

incidence of confirmed cases increased over the study period, there was significant deviation from the trend, most notably in 2014. The reasons behind this variable, but increasing, incidence of Lyme disease are likely to be multifactorial and may include raised public and practitioner awareness, variable weather patterns causing alterations in tick abundance and/or carriage of *B. burgdorferi s.l.,* and changes in human activity and behaviour.

This study observed a bimodal age distribution, with peaks at 6-10 and 61-65 years, and an overall predominance of males. This bimodal distribution has been reported in other European countries,[15–17] and matches previous UK studies.[13,14] However, the predominance of males in the current study population does not concur with other European studies, where women are over-represented.[15–17] In the USA, Lyme disease is more prevalent in males compared to females less than 60 years old, and equal or higher in women above 60 than among men.[2] In contrast, more men were hospitalised in France due to Lyme disease and more women were diagnosed by general practitioners.[38] Historically, in England and Wales, Lyme disease incidence in men and women has been similar.[13,14] The male predominance in this study may be due to the difference in health seeking behaviour between genders, with women more likely to seek healthcare at early stages of illness.[39] By presenting at later stages of Lyme disease, when pathognomonic signs may have waned, male cases may require laboratory confirmation more frequently. Further work is needed to establish the causes behind these gender differences and whether they are related to environmental or behavioral risk factors, such as occupation, leisure activities, or differences in health seeking behaviours.

There was geographical variation in Lyme disease incidence across patient residence postcode area in England and Wales, based on 58.2% of laboratory-confirmed cases. The global Moran's

For peer review only - http://bmjopen.bmj.com/site/about/guidelines.xhtml

I statistic showed that there was significant positive spatial autocorrelation, and clusters of high incidence were found in southern England. This area includes the New Forest National Park, the South Downs National Park, Salisbury Plain, Cranborne Chase Area of Outstanding Natural Beauty (AONB), Dorset AONB and Purbeck Heritage Coast. These are all popular destinations for outdoor activities and are in southern England where the Lyme disease vector *I. ricinus* is most prevalent.[1,5,40] The exposure risk from ticks is likely to be higher in these areas than other parts of the country. It is interesting that previously observed Lyme disease hotspots, such as Thetford Forest,[13] were not evident in the current study. This may be due to changing tick population dynamics and/or the prevalence of *B. burgdorferi s.l.* infection in host-seeking vectors, changing human behaviour, or the larger number of patients within this study population. It is also possible that awareness of Lyme disease is higher in these areas, and cases are successfully identified and managed in primary care without the need for serological diagnosis. Throughout the rest of England and Wales the incidence of confirmed Lyme disease cases remains relatively low (69.2% of resident postcode areas have an incidence of less than 1.0 per 100,000 population per year) compared to the majority of western Europe.[7] These data suggest that although *I. ricinus* ticks are widespread across England and Wales,[1] the proportion that carry *B. burgdorferi s.l.* is relatively low, and a higher prevalence may only exist in the tick populations in the localities highlighted. Several studies would appear to support this hypothesis,[41–43] but further work is needed to compare the incidence of human cases, abundance of ticks and prevalence of *B. burgdorferi s.l.* in ticks in the same geographic area. The areas with high incidence are predominantly rural and this is reflected in the results where the study population were disproportionately more rural compared to the national population. Information about case locality represented by PHE region is reflective of the case's referring hospital microbiology department rather than

For peer review only - http://bmjopen.bmj.com/site/about/guidelines.xhtml

the cases' residence, or location of exposure. In some instances, mainly in rural areas, this hospital may be a significant distance from the abode of the patient. This figure therefore is more reflective of the burden of Lyme disease on local microbiology departments.

Information provided at postcode area level relates to the patient's home address, and not necessarily to where the patient was bitten by a tick. Some patients are likely to have been bitten outside their resident postcode area. The further the exposure from home, the larger this spatial error will be. To date, no work has been done to quantify this error in the UK. The smoothed map (Fig 3c) attempts to account for this and shows an area of high incidence in southern-central England, centred around Southampton, Salisbury, and Weymouth and extends further west than the raw incidence data. This map highlights theoretical Lyme disease risk areas more accurately, as it accounts for the bite distance spatial error, and should be the map used for targeting public health strategies. The observed strong geographical clustering of positive cases (Fig 3d), suggests that patient residence postcode does correlate to some extent with disease risk.

This is the first time that a cohort of laboratory-confirmed Lyme disease cases across England and Wales has been described in terms of the socioeconomic status of their residential postcode area. The results suggest that patients in England diagnosed with Lyme disease are more likely to live in areas which are more affluent, have high levels of employment and education, have a higher quality of life, are less exposed to crime, but have issues with access to housing and local services. This is in contrast to the classic income gradient of health,[18–20] where the lower an individual's socioeconomic position the worse their health, but supports previous socioeconomic analyses of Lyme disease in the USA.[22,23] This study has not investigated why areas with higher socioeconomic status appear to correlate with a

For peer review only - http://bmjopen.bmj.com/site/about/guidelines.xhtml

higher incidence of Lyme disease cases but it may reflect the type of leisure activities undertaken, available leisure time, access and attitudes to the countryside by this section of society.[44] Further research is needed to better define the population of cases diagnosed with Lyme disease and why there is an association with socioeconomic status.

The only negative association with Lyme disease in England was observed for the barriers to housing and services domain and is likely due to the rural nature of the areas with the highest incidence. Rural areas score poorly as the housing tends to be expensive in relation to income and houses are a greater distance from services such as hospitals, schools and post offices. It could be reflective of this population only accessing health care, and so needing serological diagnosis, once symptoms have progressed beyond the early stages of disease. The living environment deprivation domain is a mix of housing quality, air pollution and road traffic accidents, and it is unsurprising that no association with Lyme disease incidence was observed.

In Wales, there was a significant positive correlation between case counts and the WIMD domain scores. There were an increasing number of patients living in more affluent areas. The reasons for these differences are likely to be similar to the English study population.

The main limitation of this study is the use of patient residence postcode area as a proxy both for the place where Lyme disease was acquired and the socioeconomic status of Lyme disease cases. It is unknown how representative the socioeconomic characteristics of a postcode are of individual cases. Clear socioeconomic and demographic trends and associations have been identified; however, these factors cannot be disentangled using the current datasets and so the degree of bias inherent in them is unknown. Future studies should be designed, where a

For peer review only - http://bmjopen.bmj.com/site/about/guidelines.xhtml

multivariable model can be created to identify any interaction or confounding effects of the variables under examination.

Current guidance for Lyme disease state that an erythema migrans rash is pathognomonic and further laboratory diagnostics are not required.[4] An unknown proportion of cases will be clinically diagnosed and managed in early illness by primary care clinicians and will not make it in to this dataset. Laboratory-confirmed figures will therefore underestimate the true incidence of Lyme disease seen in the general population. Without surveillance of primary care presentations, it will be hard to establish a more accurate incidence figure.

The majority of geographical data presented is reliant on case postcode data. Due to data attrition only 56.6% of cases in our dataset contained this data. Data attrition may have occurred in three ways; poor completion of the laboratory referral forms (something well documented for health professionals[45]), the non-notifiable status of clinical Lyme disease and the lack of statutory obligation to provide information about suspect cases, and the indirect route by which clinical samples are submitted for testing. Lyme disease testing is usually requested in primary care and samples are routed through hospital laboratories before reaching RIPL. There is the potential that some cases are also missed due to some laboratories (both private and public) performing their own diagnostic testing without sending samples to RIPL, as a specialist diagnostic testing laboratory, for confirmation. Testing rates may also vary in different geographies dependent upon Lyme disease awareness of health care professionals.

In this study it has been shown that laboratory-diagnosed Lyme disease cases in England and Wales have a bimodal age distribution and male predisposition. Geographical clustering of cases was seen in southern England and new insights into the socioeconomics of the resident

For peer review only - http://bmjopen.bmj.com/site/about/guidelines.xhtml

area of laboratory-confirmed Lyme disease patients were described. This study strengthens the knowledge base of Lyme disease by providing incidence maps which highlight areas where Lyme disease may place the highest burden on primary and secondary care and characterising the socio-demographics of Lyme disease cases. These data will facilitate improved public health interventions and messaging, disease surveillance, and patient management.

Acknowledgement: This work uses data provided by patients and collected by the NHS as part of their care and support and would not have been possible without access to this data. The NIHR recognises and values the role of patient data, securely accessed and stored, both in underpinning and leading to improvements in research and care.

Contributors: J.T., R.C., R.V., A.D., and J.W. contributed to the design and implementation of the research. A.S., T.B., K.R., K.H., and J.W., provided the RIPL dataset and assisted in its cleaning. J.T. performed data analysis. J.T. and J.W. wrote the manuscript in consultation with K.R., K.H., R.C., R.V., A.D., T.B., and A.S.

Funding: The research was funded by the National Institute for Health Research Health Protection Research Unit (NIHR HPRU) in Emerging and Zoonotic Infections at University of Liverpool in partnership with Public Health England (PHE), in collaboration with Liverpool School of Tropical Medicine. JT, RC, and AD are based at the University of Liverpool. TB, AS and JW are based at the PHE Rare and Imported Pathogens Laboratory, Porton Down. RV is based at PHE, Liverpool. KR and KH are based in the Emerging Infection and Zoonoses section at PHE. The views expressed are those of the authors and not necessarily those of the NHS, the NIHR, the Department of Health or Public Health England.

For peer review only - http://bmjopen.bmj.com/site/about/guidelines.xhtml

Competing Interests: None declared.

Patient consent: Not required.

Ethical approval: No ethical approval was required as these data were collected for public health surveillance under The Health Protection Legislation (England) Guidance 2010.[46]

References

1 Cull B, Pietzsch ME, Hansford KM, et al. Surveillance of British ticks: An overview of species records, host associations, and new records of Ixodes ricinus distribution. *Ticks Tick Borne Dis* 2018;9:605–14.

2 Steere AC, Strle F, Wormser GP, et al. Lyme borreliosis. *Nat Rev Dis Prim* 2016;2:16090.

3 Stanek G, Wormser GP, Gray J, et al. Lyme borreliosis. *Lancet* 2012;379:461–73.

4 National Institute for Health and Care Excellence. Lyme disease NICE guideline [NG95]. 2018.https://www.nice.org.uk/guidance/ng95 (accessed 23 Oct 2018).

5 Medlock JM, Leach SA. Effect of climate change on vector-borne disease risk in the UK. *Lancet Infect Dis* 2015;15:721–30.

6 van den Wijngaard CC, Hofhuis A, Simões M, et al. Surveillance perspective on Lyme borreliosis across the European Union and European Economic Area. *Euro Surveill* 2017;22(27).

7 Sykes RA, Makiello P. An estimate of Lyme Borreliosis incidence in Western Europe. *J Public Health (Oxf)* 2016;39:74-81

For peer review only - http://bmjopen.bmj.com/site/about/guidelines.xhtml

For peer review only

8 The Health Protection (Notification) Regulations 2010 (2010).

9 Public Health England. Lyme borreliosis epidemiology and surveillance. 2013.https://www.gov.uk/government/publications/lyme-borreliosis-epidemiology/lyme-borreliosis-epidemiology-and-surveillance (accessed 23 Oct 2018).

10 Public Health England. Infection Report Volume 11 Number 6. 2017.https://www.gov.uk/government/uploads/system/uploads/attachment_data/file/593004/hpr0617_zoos.pdf (accessed 23 Oct 2018).

11 Leeflang MM, Ang CW, Berkhout J, et al. The diagnostic accuracy of serological tests for Lyme borreliosis: a systematic review and meta-analysis. *BMC Infect Dis* 2016;16:140

12 Dessau RB, van Dam AP, Fingerle V, et al. To test or not to test? Laboratory support for the diagnosis of Lyme borreliosis. *Clin Microbiol Infect* 2018;24(2):118-124.

13 Smith R, O'Connell S, Palmer S. Lyme disease surveillance in England and Wales, 1986 1998. *Emerg Infect Dis* 2000;6(4):404–7.

14 Cooper L, Branagan-harris M, Tuson R, et al. Lyme disease and Bell's palsy: an epidemiological study of diagnosis and risk in England. *Br J Gen Pract* 2017;67(658):e329-e335.

15 Sajanti E, Virtanen M, Helve O, et al. Lyme borreliosis in Finland in 1995-2014. *Emerg Infect Dis* 2017;23:128–1288.

16 Eliassen KE, Berild D, Reiso H, et al. Incidence and antibiotic treatment of erythema migrans in Norway 2005–2009. *Ticks Tick Borne Dis* 2016;8:1-8.

For peer review only - http://bmjopen.bmj.com/site/about/guidelines.xhtml

17 Rizzoli A, Hauffe HC, Carpi G, et al. Lyme borreliosis in Europe. *Euro Surveil* 2011;16(27)

18 Hosseinpoor AR, Stewart Williams JA, Itani L, et al. Socioeconomic inequality in domains of health: results from the World Health Surveys. *BMC Public Health* 2012;12:198.

19 Pasqualini M, Lanari D, Minelli L, et al. Health and income inequalities in Europe: What is the role of circumstances? *Econ Hum Biol* 2017;26:164–73.

20 Marmot M, Friel S, Bell R, et al. Closing the gap in a generation: health equity through action on the social determinants of health. *Lancet* 2008;372:1661–9.

21 Ratnapradipa D, McDaniel JT, Barger A. Social vulnerability and Lyme disease incidence : A regional analysis of the United States, 2000-2014. *Epidemiol Biostat Public Heal* 2017;14:1–12.

22 Jackson LE, Hilborn ED, Thomas JC. Towards landscape design guidelines for reducing Lyme disease risk. *Int J Epidemiol* 2006;35:315–22.

23 Jackson LE, Levine JF, Hilborn ED. A comparison of analysis units for associating Lyme disease with forest-edge habitat. *Community Ecol* 2006;7:189–97.

24 Public Health England. Zoonoses: UK annual reports. 2017.https://www.gov.uk/government/publications/zoonoses-uk-annual-reports (accessed 23 Oct 2018).

25 Lyme disease test request form. 2017.https://www.gov.uk/government/publications/lyme-disease-test-request-form (accessed 23 Oct 2018).

For peer review only - http://bmjopen.bmj.com/site/about/guidelines.xhtml

26 Office for National Statistics - People, population and community. 2018. https://www.ons.gov.uk/peoplepopulationandcommunity. (accessed 23 Oct 2018)

27 Public Health England. Public Health England regions, local centres and emergency contacts. 2015.https://www.gov.uk/government/collections/contacts-public-health-england-regions-local-centres-and-emergency (accessed 23 Oct 2018).

28 Dudani SA. The distance-weighted k-nearest-neighbor rule. *IEEE Trans Syst Man Cybern* 1976;325–7.

29 Enas GG, Choi SC. Choice of the smoothing parameter and efficiency of k-nearest neighbor classification. *Comput Math with Appl* 1986;12:235–44.

30 Cuzick J, Edwards R. Methods for investigating localized clustering of disease. Clustering methods based on k nearest neighbour distributions. *IARC Sci Publ* 1996;135:53–67.

31 Anselin L. Local Indicators of Spatial Association—LISA. *Geogr Anal* 1995;27:93–115.

32 Anselin L, Syabri I, Smirnov O. Visualizing multivariate spatial correlation with dynamically linked windows. *New Tools for Spatial Data Analysis* 2002.

33 Gov.uk. English Indices of Deprivation 2015. 2015.https://www.gov.uk/government/statistics/english-indices-of-deprivation-2015 (accessed 23 Oct 2018).

34 Welsh Government. Welsh Index of Multiple Deprivation. 2015.http://gov.wales/statistics-and-research/welsh-index-multiple-deprivation/?lang=en (accessed 23 Oct 2018).

For peer review only

For peer review only - http://bmjopen.bmj.com/site/about/guidelines.xhtml

35 Gov.uk. 2011 Rural Urban Classification. 2013.https://www.gov.uk/government/statistics/2011-rural-urban-classification (accessed 23 Oct 2018).

36 Tulloch JSP, McGinley L, Sanchez-Vizcaino F, et al. The passive surveillance of ticks using companion animal electronic health records. *Epidemiol Infect* 2017;145:2020-2029.

37 Medlock JM, Hansford KM, Bormane A, et al. Driving forces for changes in geographical distribution of Ixodes ricinus ticks in Europe. *Parasit Vector* 2013;6:1.

38 Vandenesch A, Turbelin C, Couturier E, et al. Incidence and hospitalisation rates of Lyme borreliosis, France, 2004 to 2012. *Euro Surveill*2014;19(34)

39 Wang Y, Hunt K, Nazareth I, et al. Do men consult less than women? An analysis of routinely collected UK general practice data. *BMJ Open* 2013;3:e003320.

40 Abdullah S, Helps C, Tasker S, et al. Ticks infesting domestic dogs in the UK: a large-scale surveillance programme. *Parasit Vectors* 2016;9:391.

41 Sorouri R, Ramazani A, Karami A, et al. Isolation and characterization of Borrelia burgdorferi strains from Ixodes ricin us ticks in the southern England. *Bioimpacts* 2015;5:71–8.

42 Bettridge J, Renard M, Zhao F, et al. Distribution of Borrelia burgdorferi sensu lato in Ixodes ricinus Populations Across Central Britain. *Vector Borne Zoonotic Dis* 2013;13:139-146.

43 Hansford KM, Fonville M, Gillingham EL, et al. Ticks and Borrelia in urban and peri-urban green space habitats in a city in southern England. *Ticks Tick Borne Dis*

For peer review only - http://bmjopen.bmj.com/site/about/guidelines.xhtml

2017;8:353–61.

44 Curry N, Ravenscroft N. Countryside recreation provision in England : exploring a demand-led approach. *Land Use Policy* 2001;18:281–91.

45 Horner JS, Horner JW. Do doctors read forms? A one-year audit of medical certificates submitted to a crematorium. *J R Soc Med* 1998;91:371–6.

46 Department of Health. Health Protection Legislation (England) Guidance 2010. http://webarchive.nationalarchives.gov.uk/20130105053557/http://www.dh.gov.uk/prod_consum_dh/groups/dh_digitalassets/@dh/@en/@ps/documents/digitalasset/dh_114589.pdf (accessed 23 Oct 2018).

For peer review only

Figure Legends

Figure 1 - The annual incidence of Lyme disease in England and Wales (2013 -2016), and the number of cases per month.

Figure 2 – Population demographics of laboratory-confirmed Lyme disease cases in England and Wales, 2013-2016. (Asterisks represent age bands with a significant difference between genders. Male = Blue, Female = Red.)

Figure 3 – The average incidence (cases per 100,000 per year) of laboratory-confirmed Lyme disease in England and Wales (2013-16)

(A = Public Health England region and Wales (n = 3,985), B = Patient postcode area (n = 2,321), C = Smoothed patient postcode area, D = LISA map of significant incidence

For peer review only - http://bmjopen.bmj.com/site/about/guidelines.xhtml

clusters. Highest postcode areas and clusters are labelled accordingly; SO-Southampton, SP-Salisbury, BH-Bournemouth, RG-Reading, DT-Dorchester, GU-Guildford, TA-Taunton, TQ-Torquay, BN-Brighton, BA-Bath. Areas with no cases are labelled in red; DA-Dartford, EC-Eastern Central London, HU-Hull, and WC-Western Central London)

Figure 4 - Relationship between laboratory-confirmed Lyme disease case numbers (2013-2016) in England and the English Indices of Deprivation 2015.

For peer review only

For peer review only - http://bmjopen.bmj.com/site/about/guidelines.xhtml

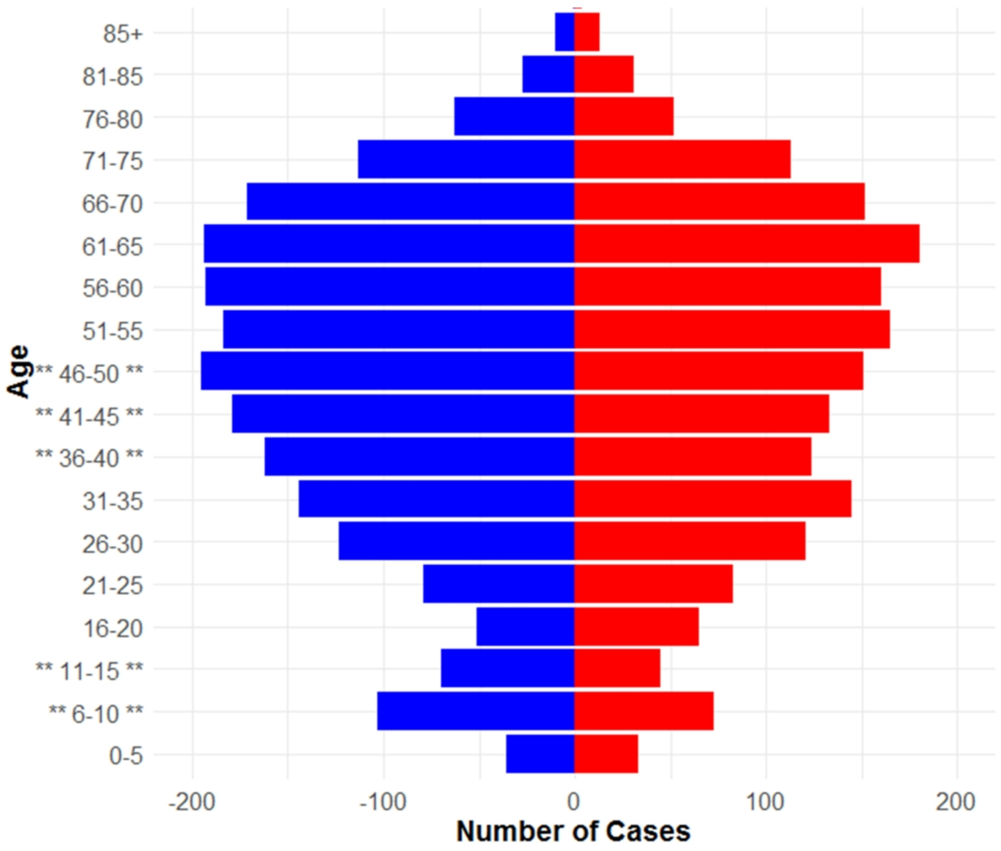

Figure 1 - The annual incidence of Lyme disease in England and Wales (2013 -2016), and the number of cases per month.

1116x939mm (96 x 96 DPI)

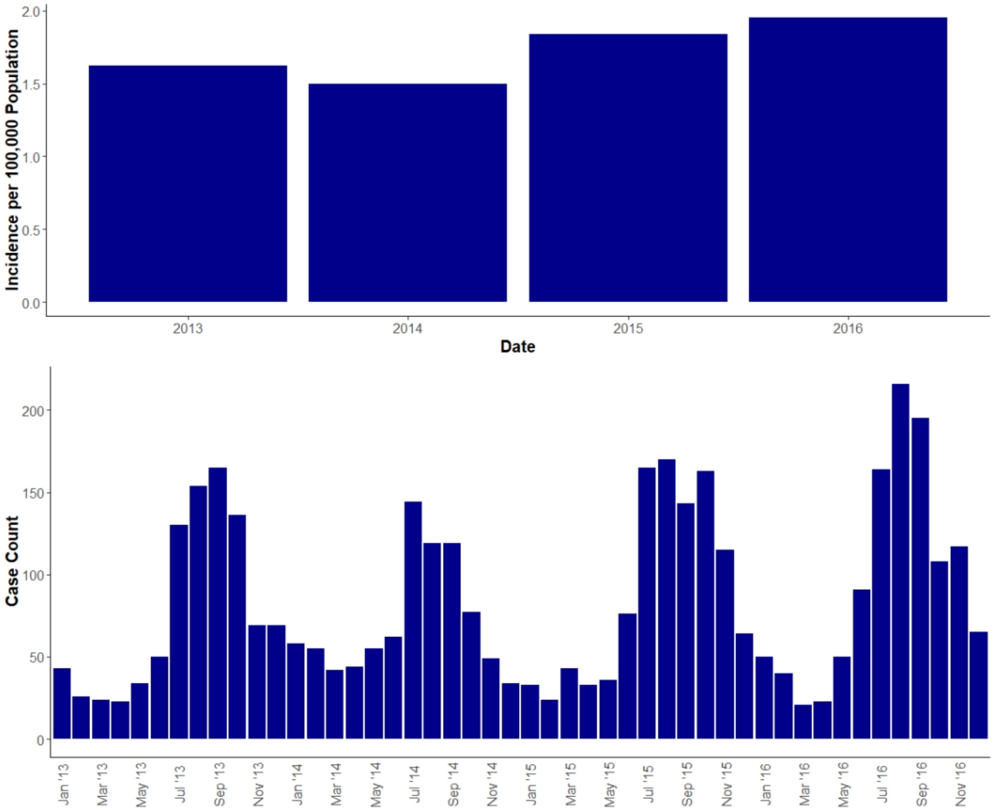

Figure 2 – Population demographics of laboratory-confirmed Lyme disease cases in England and Wales, 2013-2016. (Asterisks represent age bands with a significant difference between genders. Male = Blue, Female = Red.)

319x260mm (96 x 96 DPI)

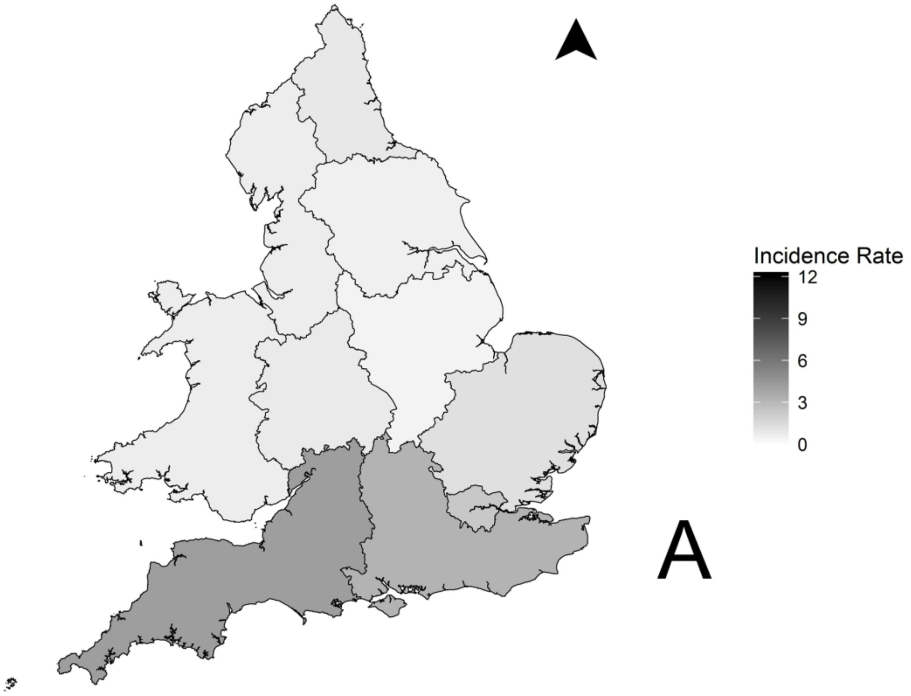

Figure 3 – The average incidence (cases per 100,000 per year) of laboratory-confirmed Lyme disease in England and Wales (2013-16) (A = Public Health England region and Wales (n = 3,985), B = Patient postcode area (n = 2,321), C = Smoothed patient postcode area, D = LISA map of significant incidence clusters. Highest postcode areas and clusters are labelled accordingly; SO-Southampton, SP-Salisbury, BH-Bournemouth, RG-Reading, DT-Dorchester, GU-Guildford, TA-Taunton, TQ-Torquay, BN-Brighton, BA-Bath. Areas with no cases are labelled in red; DA-Dartford, EC-Eastern Central London, HU-Hull, and WC-Western Central London)

172x172mm (300 x 300 DPI)

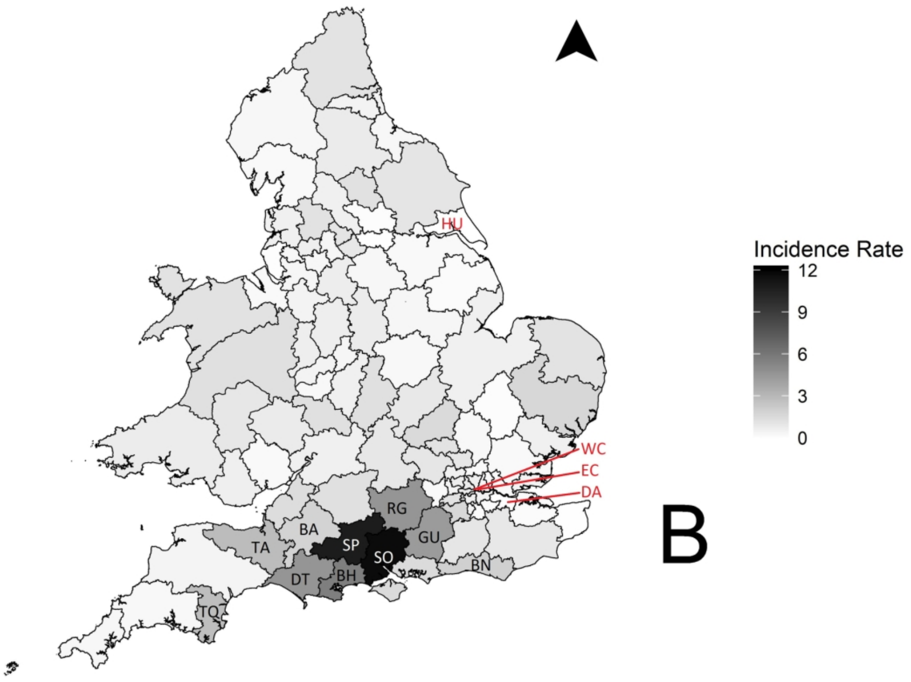

Figure 3 – The average incidence (cases per 100,000 per year) of laboratory-confirmed Lyme disease in England and Wales (2013-16) (A = Public Health England region and Wales (n = 3,985), B = Patient postcode area (n = 2,321), C = Smoothed patient postcode area, D = LISA map of significant incidence clusters. Highest postcode areas and clusters are labelled accordingly; SO-Southampton, SP-Salisbury, BH-Bournemouth, RG-Reading, DT-Dorchester, GU-Guildford, TA-Taunton, TQ-Torquay, BN-Brighton, BA-Bath. Areas with no cases are labelled in red; DA-Dartford, EC-Eastern Central London, HU-Hull, and WC-Western Central London)

172x172mm (300 x 300 DPI)

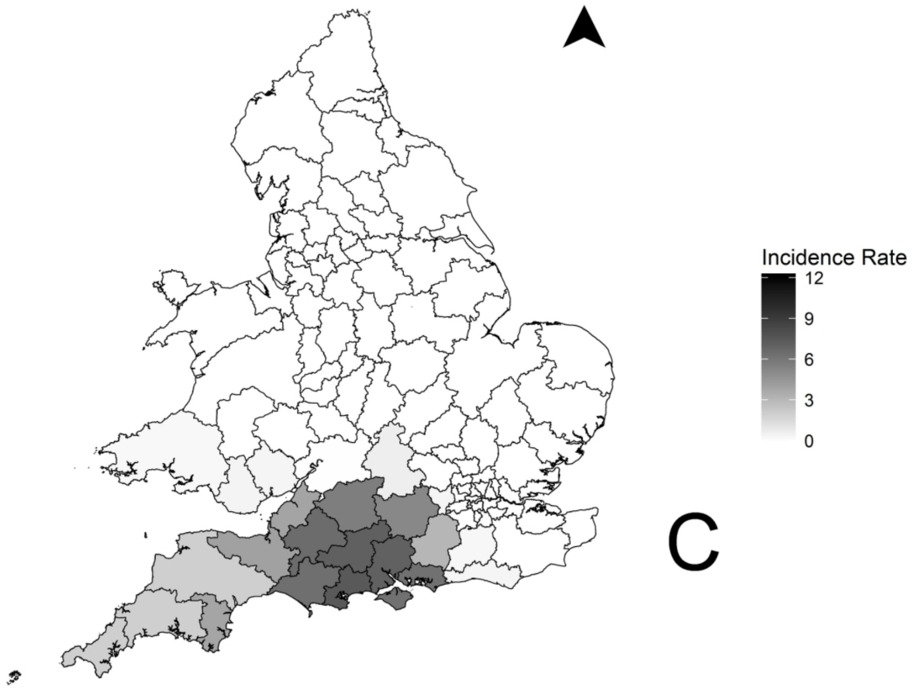

Figure 3 – The average incidence (cases per 100,000 per year) of laboratory-confirmed Lyme disease in England and Wales (2013-16) (A = Public Health England region and Wales (n = 3,985), B = Patient postcode area (n = 2,321), C = Smoothed patient postcode area, D = LISA map of significant incidence clusters. Highest postcode areas and clusters are labelled accordingly; SO-Southampton, SP-Salisbury, BH-Bournemouth, RG-Reading, DT-Dorchester, GU-Guildford, TA-Taunton, TQ-Torquay, BN-Brighton, BA-Bath. Areas with no cases are labelled in red; DA-Dartford, EC-Eastern Central London, HU-Hull, and WC-Western Central London)

177x177mm (300 x 300 DPI)

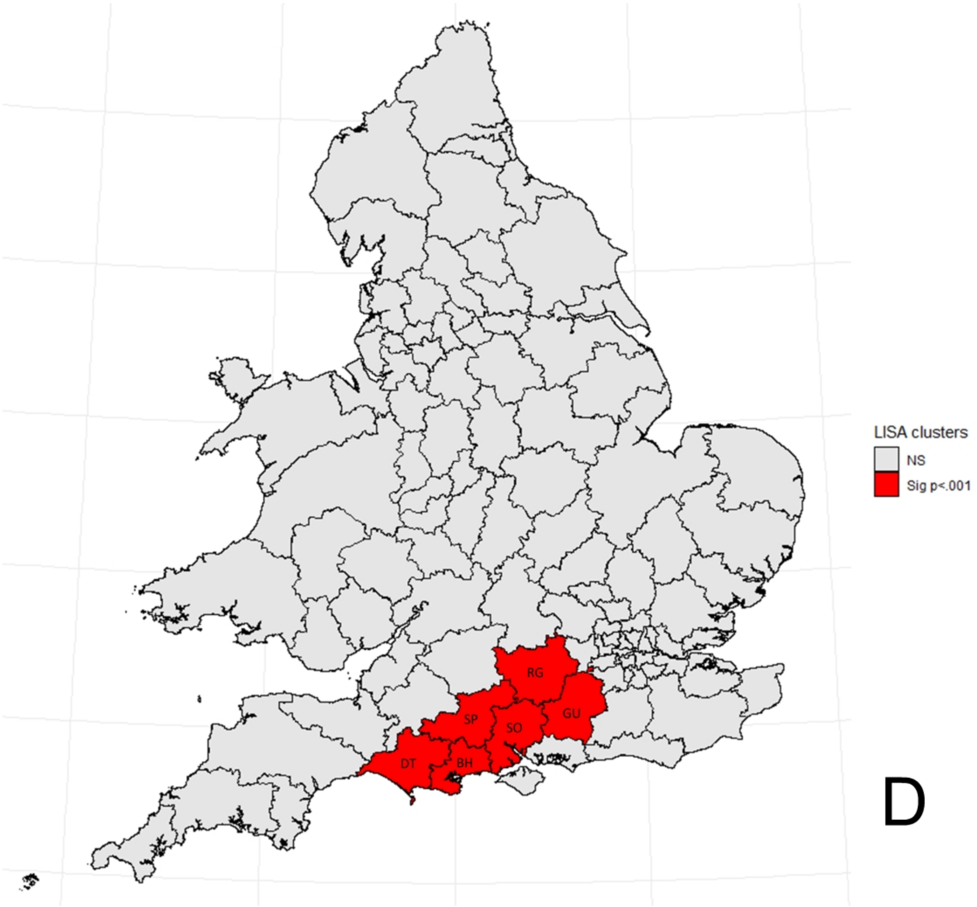

Figure 3 – The average incidence (cases per 100,000 per year) of laboratory-confirmed Lyme disease in England and Wales (2013-16)
(A = Public Health England region and Wales (n = 3,985), B = Patient postcode area (n = 2,321), C = Smoothed patient postcode area, D = LISA map of significant incidence clusters. Highest postcode areas and clusters are labelled accordingly; SO-Southampton, SP-Salisbury, BH-Bournemouth, RG-Reading, DT-Dorchester, GU-Guildford, TA-Taunton, TQ-Torquay, BN-Brighton, BA-Bath. Areas with no cases are labelled in red; DA-Dartford, EC-Eastern Central London, HU-Hull, and WC-Western Central London)

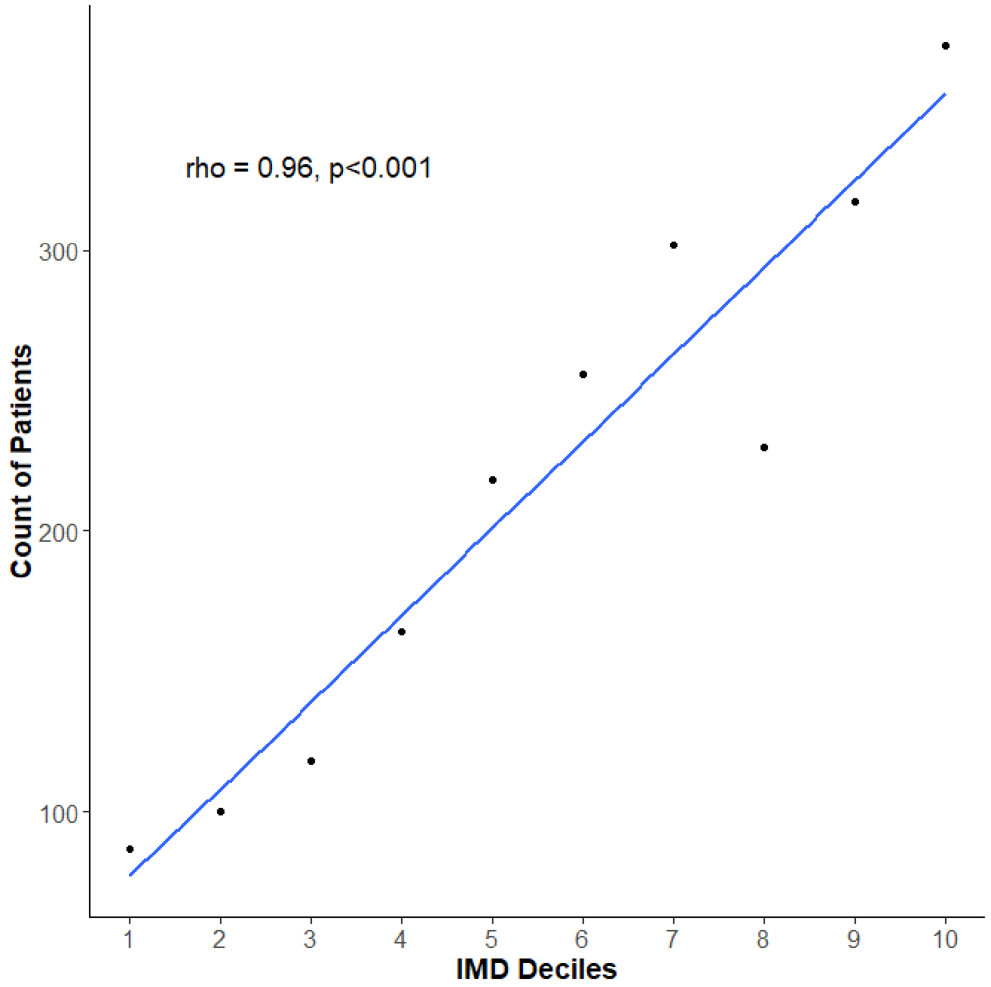

Figure 4 - Relationship between laboratory-confirmed Lyme disease case numbers (2013-2016) in England and the English Indices of Deprivation 2015.

178x178mm (96 x 96 DPI)

Supplementary Material 1

The English Indices of Deprivation (EID) ranks 32,844 geographies (Lower super output areas) containing between 1000 and 3000 population and groups these in to deciles where 1 represents the areas with the highest levels of deprivation and 10 the lowest. The Welsh Index of Multiple Deprivation (WIMD) is ranked in a similar manner but is then grouped into the following categories; the 10% with the greatest deprivation, moving up through decreasing levels of deprivation in intervals from 10-20%, 20-30%, 30-50%, and the 50% least deprived areas. The EID and WIMD categorise each geographical area with a variety of deprivation domain scores to build a summary index figure (Supplementary Table 1).

For peer review only

For peer review only - http://bmjopen.bmj.com/site/about/guidelines.xhtml

Supplementary Table 1. Summary of the English Indices of Deprivation 2015[33] and Welsh Index of Multiple Deprivation[34] domains, and their weighting to calculate an Index of Multiple Deprivation

**English Indices of Deprivation (EID) Domain**	**Description**	**Weighting for construction of Index of Multiple Deprivation (IMD)**
Income Deprivation Domain	Proportion of population experiencing deprivation due to low income	22.5%
Employment Deprivation Domain	Proportion of working age population excluded from the labor market	22.5%
Education, Skills and Training Deprivation Domain	Measures the lack of attainment and skills in the local population	13.5%
Health Deprivation and Disability Domain	Measures the risk of premature death and the impairment of quality of life through poor physical or mental health	13.5%
Crime Domain	Measures the risk of personal and material victimisation at local level	9.3%
Barriers to Housing and Services Domain	Measures the physical and financial accessibility of housing and local services (schools, supermarkets, primary care and post offices)	9.3%
Living Environment Deprivation Domain	Measures the quality of the local environment (housing, air quality and road traffic accidents)	9.3%
**Index of Multiple Deprivation (IMD)**	**Overall measure of deprivation constructed by the weighted sum of the above domains**	
**Welsh Index of Multiple Deprivation (WIMD) Domain**	**Description**	**Weighting for construction of Welsh Index of Multiple Deprivation (WIMD)**
Income Domain	Proportion of population experiencing deprivation due to low income	23.5%
Employment Domain	Proportion of working age population excluded from the labor market	23.5%
Health Domain	Measures the lack of good health	14.0%
Education Domain	Measures the extent of deprivation relating to education, training and skills	14.0%
Access to Services Domain	Measures deprivation due to a house holds inability to access services considered necessary for day to day living.	10.0%
Community Safety Domain	Measures deprivation relating to living in a safe community	5.0%
Physical Environment Domain	Measures factors in the local area that may impact on wellbeing or quality of life	5.0%
Housing Domain	Measures deprivation through lack of adequate housing	5.0%
**Welsh Index of Multiple Deprivation (WIMD)**	**Overall measure of deprivation constructed by the weighted sum of the above domains**	

For peer review only - http://bmjopen.bmj.com/site/about/guidelines.xhtml

Supplementary Material 2

Supplementary Figure - Relationship between laboratory-confirmed Lyme disease case numbers (2013-2016) in England and the component measures of the English Indices of Deprivation 2015.

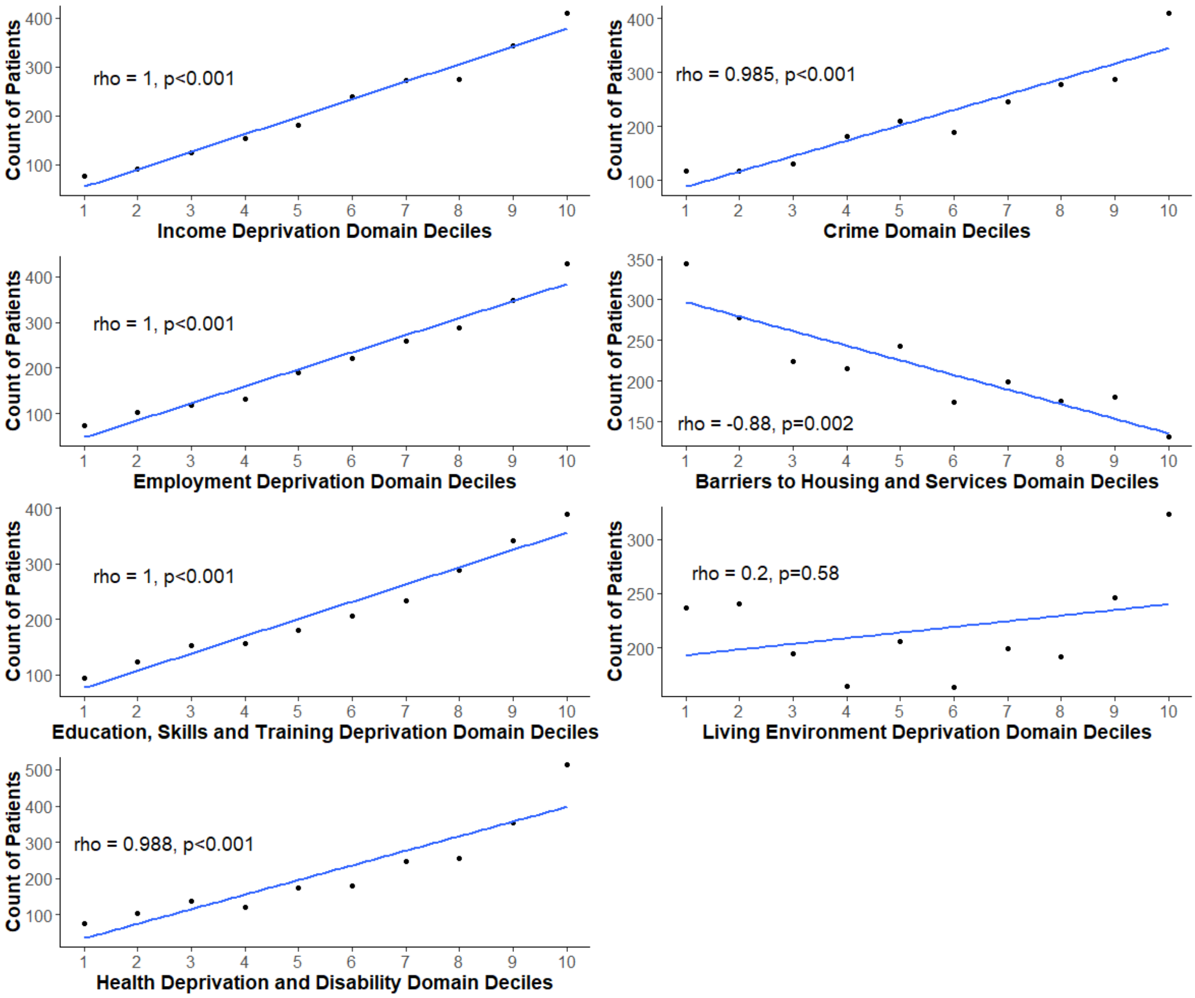

STROBE 2007 (v4) checklist of items to be included in reports of observational studies in epidemiology*

Checklist for cohort, case-control, and cross-sectional studies (combined)

Section/Topic	Item #	Recommendation	Reported on page #
**Title and abstract**	1	(*a*) Indicate the study's design with a commonly used term in the title or the abstract	2
		(*b*) Provide in the abstract an informative and balanced summary of what was done and what was found	2
**Introduction**			
Background/rationale	2	Explain the scientific background and rationale for the investigation being reported	4-6
Objectives	3	State specific objectives, including any pre-specified hypotheses	6
**Methods**			
Study design	4	Present key elements of study design early in the paper	6
Setting	5	Describe the setting, locations, and relevant dates, including periods of recruitment, exposure, follow-up, and data collection	6
Participants	6	(*a*) *Cohort study*—Give the eligibility criteria, and the sources and methods of selection of participants. Describe methods of follow-up<br>*Case-control study*—Give the eligibility criteria, and the sources and methods of case ascertainment and control selection. Give the rationale for the choice of cases and controls<br>*Cross-sectional study*—Give the eligibility criteria, and the sources and methods of selection of participants	6
		(*b*) *Cohort study*—For matched studies, give matching criteria and number of exposed and unexposed<br>*Case-control study*—For matched studies, give matching criteria and the number of controls per case	N/A
Variables	7	Clearly define all outcomes, exposures, predictors, potential confounders, and effect modifiers. Give diagnostic criteria, if applicable	6-8
Data sources/ measurement	8*	For each variable of interest, give sources of data and details of methods of assessment (measurement). Describe comparability of assessment methods if there is more than one group	6-8
Bias	9	Describe any efforts to address potential sources of bias	N/A
Study size	10	Explain how the study size was arrived at	6
Quantitative variables	11	Explain how quantitative variables were handled in the analyses. If applicable, describe which groupings were chosen and why	6-8
Statistical methods	12	(*a*) Describe all statistical methods, including those used to control for confounding	6-8
		(*b*) Describe any methods used to examine subgroups and interactions	N/A
		(*c*) Explain how missing data were addressed	N/A
		(*d*) *Cohort study*—If applicable, explain how loss to follow-up was addressed<br>*Case-control study*—If applicable, explain how matching of cases and controls was addressed	N/A

For peer review only

		Cross-sectional study—If applicable, describe analytical methods taking account of sampling strategy	
		(*e*) Describe any sensitivity analyses	N/A
**Results**			
Participants	13*	(a) Report numbers of individuals at each stage of study—eg numbers potentially eligible, examined for eligibility, confirmed eligible, included in the study, completing follow-up, and analysed	8
		(b) Give reasons for non-participation at each stage	N/A
		(c) Consider use of a flow diagram	N/A
Descriptive data	14*	(a) Give characteristics of study participants (eg demographic, clinical, social) and information on exposures and potential confounders	8-10
		(b) Indicate number of participants with missing data for each variable of interest	8-10
		(c) *Cohort study*—Summarise follow-up time (eg, average and total amount)	N/A
Outcome data	15*	*Cohort study*—Report numbers of outcome events or summary measures over time	N/A
		Case-control study—Report numbers in each exposure category, or summary measures of exposure	N/A
		Cross-sectional study—Report numbers of outcome events or summary measures	8-10
Main results	16	(*a*) Give unadjusted estimates and, if applicable, confounder-adjusted estimates and their precision (eg, 95% confidence interval). Make clear which confounders were adjusted for and why they were included	8-10
		(*b*) Report category boundaries when continuous variables were categorized	8-10
		(*c*) If relevant, consider translating estimates of relative risk into absolute risk for a meaningful time period	N/A
Other analyses	17	Report other analyses done—eg analyses of subgroups and interactions, and sensitivity analyses	8-10
**Discussion**			
Key results	18	Summarise key results with reference to study objectives	10-11
Limitations	19	Discuss limitations of the study, taking into account sources of potential bias or imprecision. Discuss both direction and magnitude of any potential bias	14-15
Interpretation	20	Give a cautious overall interpretation of results considering objectives, limitations, multiplicity of analyses, results from similar studies, and other relevant evidence	15-16
Generalisability	21	Discuss the generalisability (external validity) of the study results	15-16
**Other information**			
Funding	22	Give the source of funding and the role of the funders for the present study and, if applicable, for the original study on which the present article is based	16

*Give information separately for cases and controls in case-control studies and, if applicable, for exposed and unexposed groups in cohort and cross-sectional studies.

Note: An Explanation and Elaboration article discusses each checklist item and gives methodological background and published examples of transparent reporting. The STROBE checklist is best used in conjunction with this article (freely available on the Web sites of PLoS Medicine at http://www.plosmedicine.org/, Annals of Internal Medicine at http://www.annals.org/, and Epidemiology at http://www.epidem.com/). Information on the STROBE Initiative is available at www.strobe-statement.org.

For peer review only

BMJ Open

The demographics and geographic distribution of laboratory-confirmed Lyme disease cases in England and Wales (2013-2016): an ecological study

Journal:	*BMJ Open*
Manuscript ID	bmjopen-2018-028064.R1
Article Type:	Research
Date Submitted by the Author:	21-Jan-2019
Complete List of Authors:	Tulloch, John; University of Liverpool, NIHR Health Protection Research Unit in Emerging and Zoonotic Infections; Public Health England<br>Semper, Amanda; Public Health England, NIHR Health Protection Research Unit in Emerging and Zoonotic Infections; Public Health England, Rare and Imported Pathogens Laboratory<br>Brooks, Tim; Public Health England, NIHR Health Protection Research Unit in Emerging and Zoonotic Infections; Public Health England, Rare and Imported Pathogens Laboratory<br>Russell, Katherine; Public Health England<br>Halsby, Kate; Public Health England<br>Christley, Robert; University of Liverpool, NIHR Health Protection Research Unit in Emerging and Zoonotic Infections; University of Liverpool, Institute of Infection and Global Health<br>Radford, Alan; University of Liverpool, NIHR Health Protection Research Unit in Emerging and Zoonotic Infections; University of Liverpool, Institute of Infection and Global Health<br>Vivancos, Roberto; Public Health England, Field Epidemiology Services; Public Health England, NIHR Health Protection Research Unit in Emerging and Zoonotic Infections<br>Warner, Jenny; Public Health England, NIHR Health Protection Research Unit in Emerging and Zoonotic Infections; Public Health England, Rare and Imported Pathogens Laboratory
**Primary Subject Heading**:	Epidemiology
Secondary Subject Heading:	Infectious diseases, Public health
Keywords:	Lyme disease, Lyme borreliosis, Epidemiology < INFECTIOUS DISEASES, England, surveillance, laboratory

SCHOLARONE™
Manuscripts

Title: The demographics and geographic distribution of laboratory-confirmed Lyme disease cases in England and Wales (2013-2016): an ecological study

Authors: John S.P. Tulloch[1,2], Amanda E. Semper[3,4], Tim J.G. Brooks[3,4], Katherine Russell[5], Kate D. Halsby[5], Rob M. Christley[1,6], Alan D. Radford[1,6], Roberto Vivancos[2,7], Jenny C. Warner[3,4]

1. NIHR Health Protection Research Unit in Emerging and Zoonotic Infections, University of Liverpool, L69 3GL, UK.
2. Public Health England, Liverpool, L3 1DS, UK
3. NIHR Health Protection Research Unit in Emerging and Zoonotic Infections, Public Health England, Porton Down, SP4 0JQ, UK.
4. Rare and Imported Pathogens Laboratory, Public Health England, Porton Down, SP4 0JQ, UK
5. Public Health England, London, NW9 5EQ, UK
6. Institute of Infection and Global Health, University of Liverpool, Liverpool, CH64 7TE, UK
7. NIHR Health Protection Research Unit in Emerging and Zoonotic Infections, Public Health England, Liverpool, L3 1DS, UK.

Corresponding authors: John Tulloch; jtulloch@liverpool.ac.uk; Public Health England, Cunard Building, Water Street, Liverpool, L3 1DS, UK; +44 0344 225 0562

Jenny Warner; jenny.warner@phe.gov.uk; Rare and Imported Pathogens Laboratory, Public Health England, Porton Down, SP4 0JQ, UK; +44 01980 612899

Word count: 3571

For peer review only - http://bmjopen.bmj.com/site/about/guidelines.xhtml

Title: The demographics and geographic distribution of laboratory-confirmed Lyme disease cases in England and Wales; 2013-2016

Abstract

Objective: Lyme disease is a tick-borne disease of increasing incidence and public concern across the Northern Hemisphere. However, the socio-demographics and geographic distribution of the population affected in England and Wales are poorly understood. Therefore, the proposed study was designed to describe the demographics and distribution of laboratory-confirmed cases of Lyme disease from a national testing laboratory.

Design: An ecological study of routinely collected laboratory surveillance data

Setting: Public Health England's national Lyme disease testing laboratory.

Participants: 3,986 laboratory-confirmed cases of Lyme disease between 2013 and 2016.

Results: In England and Wales, the incidence of laboratory-confirmed Lyme disease rose significantly over the study period, from 1.62 cases per 100,000 in 2013 to 1.95 cases per 100,000 in 2016. There was a bimodal age distribution (with peaks at 6-10 and 61–65 years age bands) with a predominance of male patients. A significant clustering of areas with high Lyme disease incidence was located in southern England. An association was found between disease incidence and socioeconomic status, based on the patient's resident postcode, with more cases found in less deprived areas. Cases were disproportionately found in rural areas compared to the national population distribution.

Conclusions: These results suggest that Lyme disease patients originate from areas with higher socioeconomic status and disproportionately in rural areas. Identification of the Lyme

For peer review only - http://bmjopen.bmj.com/site/about/guidelines.xhtml

For peer review only

disease hotspots in southern England, alongside the socio-demographics described, will enable a targeted approach to public health interventions and messages.

Keywords: Lyme disease; Lyme borreliosis; epidemiology; England; Wales; surveillance; laboratory

Strengths and limitations of this study

- This study is based upon a national testing laboratory's figures and provides a much needed update on basic epidemiological information about Lyme disease in England and Wales.
- Data on the socio-economic status of Lyme disease cases is globally sparse; our findings will have implications for future public health awareness and intervention schemes and may offer new avenues for research.
- Lyme disease incidence maps have been produced to a high resolution and show significant clustering of disease; providing public health organisations with locales to target interventions.
- Geographical data, and associated variables, were based upon patient residence information rather than tick bite location.
- The study was of an ecological design and positive cases were compared to the national population, therefore no measures of risk or multivariable analysis of demographic variables were possible.

For peer review only

Introduction

Lyme disease is an important zoonotic tick-borne disease caused by spirochaetes of the *Borrelia burgdorferi sensu lato* genospecies complex. It is spread through the bites of infected *Ixodes* ticks, in the United Kingdom (UK) primarily *Ixodes ricinus.*[1] Autochthonous cases are found solely in the northern hemisphere.[2,3] Most commonly, early infection presents with an erythema migrans rash, with associated generalised flu-like symptoms.[4] Neurological manifestations, such as facial nerve palsy, can occur as part of early disseminated infection.[2] The varied presentation of the disease and the potential of increased tick exposure risk due to the extension of tick habitats as a result of changes in land management, climate and human activity, has resulted in heightened awareness and surveillance by public health organisations.[5,6]

In Western Europe the population-weighted incidence has been estimated at 22.04 cases per 100,000 person-years.[7] In the UK, Lyme disease is not a notifiable disease, but laboratory-confirmed *Borrelia* spp. are notifiable causative organisms.[8] Public Health England (PHE) compiles data on laboratory-confirmed cases of Lyme disease in England and Wales, which show a rise in the national incidence of confirmed cases from 0.38 per 100,000 population in 1997[9] to 1.95 per 100,000 population in 2016.[10] Data on laboratory-confirmed cases are provided by the national diagnostic laboratory, the PHE Rare and Imported Pathogens Laboratory (RIPL), which provides specialist advice and diagnostics for Lyme disease to the National Health Service (NHS) in England and Wales. Laboratory testing is based on serological diagnosis using a combination of screening and confirmatory immunoassays in accordance with internationally accepted best practice for Lyme disease diagnosis.[4,11,12] The incidence of cases which do not require laboratory diagnostics is unknown. These cases are most likely

For peer review only - http://bmjopen.bmj.com/site/about/guidelines.xhtml

presented to and are clinically-diagnosed and managed solely within primary care, as recommended by The National Institute for Health and Care Excellence (NICE) guidelines.[4]

Information regarding the demographics of Lyme disease cases in England and Wales is limited. Laboratory surveillance data published in 2000 describe an equal sex ratio at all ages, however, numbers were not provided and statistical comparison was not performed.[13] They describe a bimodal age distribution with peaks in childhood and at 45-64 years old. Hospital admissions data investigating Lyme disease and Bell's palsy describe a similar bimodal distribution.[14] These findings are similar to other European countries.[15–17] There is a sparsity of recent demographic data for Lyme disease in England and Wales. The geographic distribution of confirmed cases was last described in 2000.[13] They describe a tendency for cases in southern England, especially around the New Forest. However, this data may not reflect the current distribution of Lyme disease cases in England and Wales. More current data is urgently needed to enable targeted public health messaging and intervention strategies.

Globally, the negative income and education gradient of health has helped shape public health strategy and policy.[18,19] As a person's position on the socioeconomic spectrum increases, so their likelihood of better health increases. Such potentially avoidable disparities in health has led to an increased focus on understanding the social determinants of health[20] and developing measures to address these. Work to explore the association between socioeconomic status and Lyme disease incidence is limited. In the United States of America (USA) persons were to found to be at greatest risk of Lyme disease if they lived in the highest or lowest socially vulnerable areas.[21] Two studies found a relationship between Lyme disease incidence and median annual household income, with incidence peaking at around

For peer review only - http://bmjopen.bmj.com/site/about/guidelines.xhtml

80,000 USD.[22,23] However, a consistent relationship between the socioeconomic state of an individual and their Lyme disease acquisition risk has yet to emerge. In particular, no in-depth research has been performed in Europe investigating the socioeconomics of the Lyme disease patient cohort.

The aim of this study was to utilise information collected through routine surveillance in England and Wales to describe the demographics and geographic distribution of laboratory-confirmed Lyme disease cases over a four year period. Correlations between Lyme disease incidence and socioeconomic indices were analysed, using patient residence postcode as a proxy for individual patient characteristics. New insight will be provided into the key demographic, geographical and social determinants of the Lyme disease patient population. This would allow us to identify potentially at-risk populations, shape public health interventions and assist in appropriate disease awareness.

Methods

A retrospective analysis was performed using data extracted from the PHE Rare and Imported Pathogens Laboratory's (RIPL) laboratory information management system (LIMS), between 1st January 2013 and 31st December 2016, for laboratory-confirmed Lyme disease cases, the same data as used for PHE's Zoonoses Report.[24] The RIPL LIMS contains information provided on the Lyme disease referral form submitted at the time of sample submission and any additional information provided by clinicians during case follow up and management.[25] The form captures information on the age, gender, location, clinical symptoms and travel history of the patient. Data were cleaned and duplicate (across all variable) records were removed where necessary.

For peer review only - http://bmjopen.bmj.com/site/about/guidelines.xhtml

Annual Lyme disease incidence estimates were calculated, using the Office for National Statistics (ONS) mid-year population estimates as the denominator population.[26] A Chi-squared test for trend and a Chi-squared test for departure from the trend were used to analyse trends in incidence. Cases were stratified by age and gender. Using binomial tests, the null hypothesis that there was no difference in case numbers between males and females was tested within differing age bands, and overall.

Geographical information was collated based on (1) the regional origin of a diagnostic sample (usually a hospital microbiology department) consisting of eight PHE regions, and Wales as a whole,[27] and (2) the postcode area of the patient. These were used to calculate average annual incidence for the study period. To account for the unknown distance between a patient's home address and where they were bitten and to highlight any disease hotspots, the disease incidence map for postcode area was smoothed. A k-nearest neighbours (k-NN) approach was used.[28–30] In this approach, a Queen contiguity was used to define geographical neighbours, this defines a neighbour as being an area that shares a common edge or vertex. k is defined as the number of neighbours used for smoothing. k is equal to the square root of the total number of discrete geographical areas rounded to the nearest whole odd number (i.e. 105 postcode areas, its square root being 10.2, therefore k=11). Exploratory spatial data analysis (EDSA)[31,32] was used to explore the spatial autocorrelation of the postcode area incidence map. Global and local Moran's I values were calculated, and a LISA (Local Indicators of Spatial Association) significance map constructed to highlight any significant clusters. In both the k-NN smoothing and Moran's I calculations, a queen adjacency matrix was used.

For peer review only - http://bmjopen.bmj.com/site/about/guidelines.xhtml

Patient postcode was linked to ONS socioeconomic data,[26] enabling a description of the socioeconomic characteristics of the population in which a Lyme disease case was resident. If no patient postcode was recorded, these cases were excluded from the analysis. Socioeconomic status is reported through the English Indices of Deprivation (EID) 2015[33] and the Welsh Index of Multiple Deprivation (WIMD) 2014[34] (Supplementary Material 1, Table 1). Postcode area case count data were matched independently to the EID and WIMD, and a rural urban classification. As EID and WIMD are on a discrete ordinal scale, Spearman's rank correlation was used to calculate the correlation between the number of cases and deprivation score. The proportion of cases with their home addresses located in either a rural or urban area, were compared to the national rural urban classification from the ONS.[35] This was performed using a Chi-squared test of independence for both English and Welsh data.

All statistical and spatial analyses were carried out using R language (version 3.2.0) (R Core Team 2015). Results were deemed significant where $p<0.05$.

Patient and public involvement

The public or patients were not involved in the development of the research question or the outcome measures. However, this research was informed by the research recommendations in the 2018 Lyme disease NICE guidelines,[4] which had patient and public involvement. Investigators have and will continue to present these findings at regional and national events and to the general public, patients groups, NHS organisations, public health departments and governments agencies.

Results

For peer review only - http://bmjopen.bmj.com/site/about/guidelines.xhtml

In total 3,986 unique cases, 3,893 cases in England and 93 in Wales, meeting a serological diagnosis of Lyme disease were identified in the RIPL LIMS between 1st January 2013 and the 31st December 2016. Of these, 98.7% (n=3,935) had complete records for date of submission, gender and age. Only 10.5% (n=417) of cases had details on the submission form confirming or excluding international travel from a case's clinical history. Due to the low completeness of this variable, it was concluded that further analysis of travel history would not be performed.

The annual incidence of laboratory-confirmed Lyme disease cases in England and Wales rose from 1.62 per 100,000 population in 2013, to 1.95 in 2016. These figures are identical to PHE's official incidence figures as they used the same data source.[10] There was evidence of an overall association between incidence and year (χ^2=43.13, p<0.001). This association took the form of a trend with increasing incidence each year (χ^2=30.17, p<0.001). Departures from the trend were significant (χ^2=43.1-30.1=12.96, p<0.001), as shown by the fall in incidence in 2014. There was marked seasonality, with the peak numbers of cases being diagnosed in the summer months each year (Fig 1).

Across all ages there were significantly more male (n=2,096) than female (n=1,839) cases (p < 0.001), with a bimodal age distribution, with peaks at 6-10 and 61-65 year age bands (Fig 2). Grouping the data in 5-year age bands, there were significantly more men than women in the 6-10 (p=0.03), 11-15 (p=0.03), 36-40 (p=0.01), 41-45 (p=0.02), and 46-50 (p=0.04) age groups.

Data were available about PHE regions for 99.9% (n=3,985) of the study population (Fig 3a). The patient residence postcode was not provided on 1,665 of the referral forms, and therefore only 58.2% (n=2,321) of cases could be described at postcode area resolution. The average percentage of missing postcode data by PHE region was 31.9% (range: 10.8%-76.1%). The regions with the highest missing postcode data were London (76.1%), South West

For peer review only - http://bmjopen.bmj.com/site/about/guidelines.xhtml

(49.4%), and North West (44.7%). The regions with the lowest missing postcode data were Wales (10.8%), North East (12.1%), and West Midlands (14.5%). The South West PHE region had the highest incidence of Lyme disease in England and Wales; none of the PHE regions, nor Wales, reported zero cases. The postcode areas with the highest average annual incidence of Lyme disease were Southampton (11.65 cases per 100,000 per year), Salisbury (10.75), Bournemouth (5.62), Reading (4.59), Dorchester (4.57), Guildford (4.31), Taunton (2.79), Torquay (2.75), Brighton (1.96), and Bath (1.84) (Fig 3b). These areas are all in southern England. Only four postcode areas had no laboratory-confirmed cases in the four year surveillance period (Fig 3b), namely Dartford, Eastern Central London, Hull, and Western Central London. The smoothed data showed a trend for the areas of highest incidence to be located in southern-central England (Fig 3c). There was significant spatial autocorrelation, the global Moran's I was 0.564 ($p=0.01$), indicating that postcode areas with similar incidence are clustered together. LISA mapping identified six areas as significant clusters of high incidence (Fig 3d); Southampton, Salisbury, Bournemouth, Reading, Dorchester, and Guildford (for all $p<0.001$).

Using patient residence postcode data, it was possible to match 55.6% (n=2,165) of English records to the English Indices of Deprivation and 98.2% (n=92) of Welsh records to the Welsh Index of Multiple Deprivation (WIMD). An overall significant positive correlation between the number of cases and Index of Multiple Deprivation (IMD) decile was observed ($\rho=0.96$, $p<0.001$), with more Lyme disease cases found in less deprived areas (Fig 4). This significant positive correlation was seen across all domains of deprivation, except the 'Barriers to Housing and Services Domain' where this trend was reversed ($\rho=-0.88$, $p=0.002$) and the 'Living Environment Deprivation Domain' where there was no significant correlation ($\rho=0.2$, $p=0.58$) (Supplementary material 2). An overall significant positive correlation between the number of cases and WIMD rank was observed ($\rho=0.89$, $p=0.04$), with more Lyme disease cases found in the least deprived areas.

For peer review only - http://bmjopen.bmj.com/site/about/guidelines.xhtml

When compared to the national population, the study population was disproportionately more likely to live in a rural area, for both English (p<0.001) and Welsh (p<0.001) sections of the study population (Table 1).

Table 1 – The rural urban classification of laboratory-confirmed cases of Lyme disease in England and Wales (2013-2016) compared to the national census population

Category	Percentage of English Study Population	Percentage of Welsh Study Population	Percentage of 2015 census population
Rural	34.3% (n=743)	47.8% (n=44)	17.9%
Urban	65.7% (n=1,422)	52.2% (n=48)	82.1%

Discussion

Between 2013 and 2016 there was a significant increase in annual incidence of cases of confirmed Lyme disease, with a seasonality that matched previous publications and has been well documented.[9] The observed seasonality closely matches *I. ricinus* tick population dynamics in the UK, which annually peak around June and July.[1,36] Concerns have been raised about how the expansion of tick habitats due to changes in land use and management, and climate change, may be increasing the risk of Lyme disease infection.[5,37] Although the incidence of confirmed cases increased over the study period, there was significant deviation from the trend, most notably in 2014. The reasons behind this variable, but increasing, incidence of Lyme disease are likely to be multifactorial and may include raised public and practitioner awareness, variable weather patterns causing alterations in tick abundance and/or carriage of *B. burgdorferi s.l.,* and changes in human activity and behaviour.

This study observed a bimodal age distribution, with peaks at 6-10 and 61-65 years, and an overall predominance of males. This bimodal distribution has been reported in other

For peer review only - http://bmjopen.bmj.com/site/about/guidelines.xhtml

European countries,[15–17] and matches previous UK studies.[13,14] However, the predominance of males in the current study population does not concur with other European studies, where women are over-represented.[15–17] In the USA, Lyme disease is more prevalent in males compared to females less than 60 years old, and equal or higher in women above 60 than among men.[2] In contrast, more men were hospitalised in France due to Lyme disease and more women were diagnosed by general practitioners.[38] Historically, in England and Wales, Lyme disease incidence in men and women has been similar.[13,14] The male predominance in this study may be due to the difference in health seeking behaviour between genders, with women more likely to seek healthcare at early stages of illness.[39] By presenting at later stages of Lyme disease, when pathognomonic signs may have waned, male cases may require laboratory confirmation more frequently. Further work is needed to establish the causes behind these gender differences and whether they are related to environmental or behavioral risk factors, such as occupation, leisure activities, or differences in health seeking behaviours.

There was geographical variation in Lyme disease incidence across patient residence postcode area in England and Wales, based on 58.2% of laboratory-confirmed cases. The global Moran's I statistic showed that there was significant positive spatial autocorrelation, and clusters of high incidence were found in southern England. This area includes the New Forest National Park, the South Downs National Park, Salisbury Plain, Cranborne Chase Area of Outstanding Natural Beauty (AONB), Dorset AONB and Purbeck Heritage Coast. These are all popular destinations for outdoor activities and are in southern England where the Lyme disease vector *I. ricinus* is most prevalent.[1,5,40] The exposure risk from ticks is likely to be higher in these areas than other parts of the country. It is interesting that previously observed Lyme disease hotspots, such as Thetford Forest,[13] were not evident in the current study. This may be due

For peer review only - http://bmjopen.bmj.com/site/about/guidelines.xhtml

to changing tick population dynamics and/or the prevalence of *B. burgdorferi s.l.* infection in host-seeking vectors, changing human behaviour, or the larger number of patients within this study population. It is also possible that awareness of Lyme disease is higher in these areas, and cases are successfully identified and managed in primary care without the need for serological diagnosis. Throughout the rest of England and Wales the incidence of confirmed Lyme disease cases remains relatively low (69.2% of resident postcode areas have an incidence of less than 1.0 per 100,000 population per year) compared to the majority of western Europe.[7] The four postcode areas with no laboratory-confirmed cases were all surrounded by areas with very low incidence and is likely to be reflective of the overall low incidence of Lyme disease in England and Wales. Although *I. ricinus* ticks are widespread across England and Wales,[1] the risk of contracting Lyme disease appears to be relatively low. It is possible that the tick populations found within high Lyme disease incidence areas may also have the highest *B.burgdorferi s.l.* prevalence. Several studies would appear to support this hypothesis,[41–43] but further work is needed to compare the incidence of human cases, abundance of ticks and prevalence of *B. burgdorferi s.l.* in ticks in the same geographic area. The areas with high incidence are predominantly rural and this is reflected in the results where the study population were disproportionately more rural compared to the national population. Information about case locality represented by PHE region is reflective of the case's referring hospital microbiology department rather than the cases' residence, or location of exposure. In some instances, mainly in rural areas, this hospital may be a significant distance from the abode of the patient. This figure therefore is more reflective of the burden of Lyme disease on local microbiology departments.

Information provided at postcode area level relates to the patient's home address, and not necessarily to where the patient was bitten by a tick. Some patients are likely to have been

For peer review only - http://bmjopen.bmj.com/site/about/guidelines.xhtml

bitten outside their resident postcode area. The further the exposure from home, the larger this spatial error will be. To date, no work has been done to quantify this error in the UK. The smoothed map (Fig 3c) attempts to account for this and shows an area of high incidence in southern-central England, centred around Southampton, Salisbury, and Weymouth and extends further west than the raw incidence data. This map highlights theoretical Lyme disease risk areas more accurately, as it accounts for the bite distance spatial error, and should be the map used for targeting public health strategies. The observed strong geographical clustering of positive cases (Fig 3d), suggests that patient residence postcode does correlate to some extent with disease risk.

This is the first time that a cohort of laboratory-confirmed Lyme disease cases across England and Wales has been described in terms of the socioeconomic status of their residential postcode area. The results suggest that patients in England diagnosed with Lyme disease are more likely to live in areas which are more affluent, have high levels of employment and education, have a higher quality of life, are less exposed to crime, but have issues with access to housing and local services. This is in contrast to the classic income gradient of health,[18–20] where the lower an individual's socioeconomic position the worse their health, but supports previous socioeconomic analyses of Lyme disease in the USA.[22,23] This study has not investigated why areas with higher socioeconomic status appear to correlate with a higher incidence of Lyme disease cases but it may reflect the type of leisure activities undertaken, available leisure time, access and attitudes to the countryside by this section of society.[44] Further research is needed to better define the population of cases diagnosed with Lyme disease and why there is an association with socioeconomic status.

For peer review only - http://bmjopen.bmj.com/site/about/guidelines.xhtml

For peer review only

The only negative association with Lyme disease in England was observed for the barriers to housing and services domain and is likely due to the rural nature of the areas with the highest incidence. Rural areas score poorly as the housing tends to be expensive in relation to income and houses are a greater distance from services such as hospitals, schools and post offices. It could be reflective of this population only accessing health care, and so needing serological diagnosis, once symptoms have progressed beyond the early stages of disease. The living environment deprivation domain is a mix of housing quality, air pollution and road traffic accidents, and it is unsurprising that no association with Lyme disease incidence was observed.

In Wales, there was a significant positive correlation between case counts and the WIMD domain scores. There were an increasing number of patients living in more affluent areas. The reasons for these differences are likely to be similar to the English study population.

The main limitation of this study is the use of patient residence postcode area as a proxy both for the place where Lyme disease was acquired and the socioeconomic status of Lyme disease cases. It is unknown how representative the socioeconomic characteristics of a postcode are of individual cases. Clear socioeconomic and demographic trends and associations have been identified; however, these factors cannot be disentangled using the current datasets and so the degree of bias inherent in them is unknown. Future studies should be designed, where a multivariable model can be created to identify any interaction or confounding effects of the variables under examination.

Current guidance for Lyme disease state that an erythema migrans rash is pathognomonic and further laboratory diagnostics are not required.[4] An unknown proportion of cases will be clinically diagnosed and managed in early illness by primary care clinicians and will not

For peer review only - http://bmjopen.bmj.com/site/about/guidelines.xhtml

make it in to this dataset. Laboratory-confirmed figures will therefore underestimate the true incidence of Lyme disease seen in the general population. Without surveillance of primary care presentations, it will be hard to establish a more accurate incidence figure.

The majority of geographical data presented is reliant on case postcode data. Due to data attrition only 58.2% of cases in our dataset contained this data. Data attrition may have occurred in three ways; poor completion of the laboratory referral forms (something well documented for health professionals[45]), the non-notifiable status of clinical Lyme disease and the lack of statutory obligation to provide information about suspect cases, and the indirect route by which clinical samples are submitted for testing. Lyme disease testing is usually requested in primary care and samples are routed through hospital laboratories before reaching RIPL. There is the potential that some cases are also missed due to some laboratories (both private and public) performing their own diagnostic testing without sending samples to RIPL, as a specialist diagnostic testing laboratory, for confirmation. Testing rates may also vary in different geographies dependent upon Lyme disease awareness of health care professionals. The results indicated that the degree of missingness was not even across all PHE regions. This level of missingness had not been anticipated, and there is the potential for bias within the results. It would be possible to extract missing geographical data by linking cases to datasets with patient postcode data, via a unique patient identifier (NHS Number). However, data linkage for this dataset was not possible as part of public health surveillance under The Health Protection Legislation (England) Guidance 2010.[46] These geographical results should be interpreted within the above context and with an appropriate level of prudence.

For peer review only - http://bmjopen.bmj.com/site/about/guidelines.xhtml

For peer review only

In this study it has been shown that laboratory-diagnosed Lyme disease cases in England and Wales have a bimodal age distribution and male predisposition. Geographical clustering of cases was seen in southern England and new insights into the socioeconomics of the resident area of laboratory-confirmed Lyme disease patients were described. This study strengthens the knowledge base of Lyme disease by providing incidence maps which highlight areas where Lyme disease may place the highest burden on primary and secondary care and characterising the socio-demographics of Lyme disease cases. These data will facilitate improved public health interventions and messaging, disease surveillance, and patient management.

Acknowledgement: This work uses data provided by patients and collected by the NHS as part of their care and support and would not have been possible without access to this data. The NIHR recognises and values the role of patient data, securely accessed and stored, both in underpinning and leading to improvements in research and care.

Contributors: J.T., R.C., R.V., A.D., and J.W. contributed to the design and implementation of the research. A.S., T.B., K.R., K.H., and J.W., provided the RIPL dataset and assisted in its cleaning. J.T. performed data analysis. J.T. and J.W. wrote the manuscript in consultation with K.R., K.H., R.C., R.V., A.D., T.B., and A.S.

Funding: The research was funded by the National Institute for Health Research Health Protection Research Unit (NIHR HPRU) in Emerging and Zoonotic Infections at University of Liverpool in partnership with Public Health England (PHE), in collaboration with Liverpool School of Tropical Medicine. JT, RC, and AD are based at the University of Liverpool. TB, AS and JW are based at the PHE Rare and Imported Pathogens Laboratory, Porton Down. RV is based at PHE, Liverpool. KR and KH are based in the Emerging Infection and Zoonoses section

For peer review only - http://bmjopen.bmj.com/site/about/guidelines.xhtml

at PHE. The views expressed are those of the authors and not necessarily those of the NHS, the NIHR, the Department of Health or Public Health England.

Competing Interests: None declared.

Patient consent: Not required.

Ethical approval: No ethical approval was required as these anonymised patient data were collected for public health surveillance under The Health Protection Legislation (England) Guidance 2010.[46]

Data sharing statement: The data that support the findings of this study are available from Public Health England, but restrictions apply to the availability of these data as they are not publicly available. Aggregated data may be available from Public Health England on reasonable request and with permission of Public Health England; subject to the requirement of the General Data Protection Regulation.

References

1 Cull B, Pietzsch ME, Hansford KM, et al. Surveillance of British ticks: An overview of species records, host associations, and new records of Ixodes ricinus distribution. *Ticks Tick Borne Dis* 2018;9:605–14.

2 Steere AC, Strle F, Wormser GP, et al. Lyme borreliosis. *Nat Rev Dis Prim* 2016;2:16090.

3 Stanek G, Wormser GP, Gray J, et al. Lyme borreliosis. *Lancet* 2012;379:461–73.

4 National Institute for Health and Care Excellence. Lyme disease NICE guideline [NG95]. 2018.https://www.nice.org.uk/guidance/ng95 (accessed 23 Oct 2018).

For peer review only - http://bmjopen.bmj.com/site/about/guidelines.xhtml

5 Medlock JM, Leach SA. Effect of climate change on vector-borne disease risk in the UK. *Lancet Infect Dis* 2015;15:721–30.

6 van den Wijngaard CC, Hofhuis A, Simões M, et al. Surveillance perspective on Lyme borreliosis across the European Union and European Economic Area. *Euro Surveill* 2017;22(27).

7 Sykes RA, Makiello P. An estimate of Lyme Borreliosis incidence in Western Europe. *J Public Health (Oxf)* 2016;39:74-81

8 The Health Protection (Notification) Regulations 2010 (2010).

9 Public Health England. Lyme borreliosis epidemiology and surveillance. 2013.https://www.gov.uk/government/publications/lyme-borreliosis-epidemiology/lyme-borreliosis-epidemiology-and-surveillance (accessed 23 Oct 2018).

10 Public Health England. Infection Report Volume 11 Number 6. 2017.https://www.gov.uk/government/uploads/system/uploads/attachment_data/file/593004/hpr0617_zoos.pdf (accessed 23 Oct 2018).

11 Leeflang MM, Ang CW, Berkhout J, et al. The diagnostic accuracy of serological tests for Lyme borreliosis: a systematic review and meta-analysis. *BMC Infect Dis* 2016;16:140

12 Dessau RB, van Dam AP, Fingerle V, et al. To test or not to test? Laboratory support for the diagnosis of Lyme borreliosis. *Clin Microbiol Infect* 2018;24(2):118-124.

13 Smith R, O'Connell S, Palmer S. Lyme disease surveillance in England and Wales, 1986 1998. *Emerg Infect Dis* 2000;6(4):404–7.

For peer review only - http://bmjopen.bmj.com/site/about/guidelines.xhtml

14 Cooper L, Branagan-harris M, Tuson R, et al. Lyme disease and Bell's palsy: an epidemiological study of diagnosis and risk in England. *Br J Gen Pract* 2017;67(658):e329-e335.

15 Sajanti E, Virtanen M, Helve O, et al. Lyme borreliosis in Finland in 1995-2014. *Emerg Infect Dis* 2017;23:128–1288.

16 Eliassen KE, Berild D, Reiso H, et al. Incidence and antibiotic treatment of erythema migrans in Norway 2005–2009. *Ticks Tick Borne Dis* 2016;8:1-8.

17 Rizzoli A, Hauffe HC, Carpi G, et al. Lyme borreliosis in Europe. *Euro Surveil* 2011;16(27)

18 Hosseinpoor AR, Stewart Williams JA, Itani L, et al. Socioeconomic inequality in domains of health: results from the World Health Surveys. *BMC Public Health* 2012;12:198.

19 Pasqualini M, Lanari D, Minelli L, et al. Health and income inequalities in Europe: What is the role of circumstances? *Econ Hum Biol* 2017;26:164–73.

20 Marmot M, Friel S, Bell R, et al. Closing the gap in a generation: health equity through action on the social determinants of health. *Lancet* 2008;372:1661–9.

21 Ratnapradipa D, McDaniel JT, Barger A. Social vulnerability and Lyme disease incidence : A regional analysis of the United States, 2000-2014. *Epidemiol Biostat Public Heal* 2017;14:1–12.

22 Jackson LE, Hilborn ED, Thomas JC. Towards landscape design guidelines for reducing Lyme disease risk. *Int J Epidemiol* 2006;35:315–22.

For peer review only - http://bmjopen.bmj.com/site/about/guidelines.xhtml

23 Jackson LE, Levine JF, Hilborn ED. A comparison of analysis units for associating Lyme disease with forest-edge habitat. *Community Ecol* 2006;7:189–97.

24 Public Health England. Zoonoses: UK annual reports. 2017.https://www.gov.uk/government/publications/zoonoses-uk-annual-reports (accessed 23 Oct 2018).

25 Lyme disease test request form. 2017.https://www.gov.uk/government/publications/lyme-disease-test-request-form (accessed 23 Oct 2018).

26 Office for National Statistics - People, population and community. 2018. https://www.ons.gov.uk/peoplepopulationandcommunity. (accessed 23 Oct 2018)

27 Public Health England. Public Health England regions, local centres and emergency contacts. 2015.https://www.gov.uk/government/collections/contacts-public-health-england-regions-local-centres-and-emergency (accessed 23 Oct 2018).

28 Dudani SA. The distance-weighted k-nearest-neighbor rule. *IEEE Trans Syst Man Cybern* 1976;325–7.

29 Enas GG, Choi SC. Choice of the smoothing parameter and efficiency of k-nearest neighbor classification. *Comput Math with Appl* 1986;12:235–44.

30 Cuzick J, Edwards R. Methods for investigating localized clustering of disease. Clustering methods based on k nearest neighbour distributions. *IARC Sci Publ* 1996;135:53–67.

31 Anselin L. Local Indicators of Spatial Association—LISA. *Geogr Anal* 1995;27:93–115.

For peer review only - http://bmjopen.bmj.com/site/about/guidelines.xhtml

32 Anselin L, Syabri I, Smirnov O. Visualizing multivariate spatial correlation with dynamically linked windows. *New Tools for Spatial Data Analysis* 2002.

33 Gov.uk. English Indices of Deprivation 2015. 2015.https://www.gov.uk/government/statistics/english-indices-of-deprivation-2015 (accessed 23 Oct 2018).

34 Welsh Government. Welsh Index of Multiple Deprivation. 2015.http://gov.wales/statistics-and-research/welsh-index-multiple-deprivation/?lang=en (accessed 23 Oct 2018).

35 Gov.uk. 2011 Rural Urban Classification. 2013.https://www.gov.uk/government/statistics/2011-rural-urban-classification (accessed 23 Oct 2018).

36 Tulloch JSP, McGinley L, Sanchez-Vizcaino F, et al. The passive surveillance of ticks using companion animal electronic health records. *Epidemiol Infect* 2017;145:2020-2029.

37 Medlock JM, Hansford KM, Bormane A, et al. Driving forces for changes in geographical distribution of Ixodes ricinus ticks in Europe. *Parasit Vector* 2013;6:1.

38 Vandenesch A, Turbelin C, Couturier E, et al. Incidence and hospitalisation rates of Lyme borreliosis, France, 2004 to 2012. *Euro Surveill*2014;19(34)

39 Wang Y, Hunt K, Nazareth I, et al. Do men consult less than women? An analysis of routinely collected UK general practice data. *BMJ Open* 2013;3:e003320.

40 Abdullah S, Helps C, Tasker S, et al. Ticks infesting domestic dogs in the UK: a large-scale surveillance programme. *Parasit Vectors* 2016;9:391.

For peer review only - http://bmjopen.bmj.com/site/about/guidelines.xhtml

41 Sorouri R, Ramazani A, Karami A, et al. Isolation and characterization of Borrelia burgdorferi strains from Ixodes ricin us ticks in the southern England. *Bioimpacts* 2015;5:71–8.

42 Bettridge J, Renard M, Zhao F, et al. Distribution of Borrelia burgdorferi sensu lato in Ixodes ricinus Populations Across Central Britain. *Vector Borne Zoonotic Dis* 2013;13:139-146.

43 Hansford KM, Fonville M, Gillingham EL, et al. Ticks and Borrelia in urban and peri-urban green space habitats in a city in southern England. *Ticks Tick Borne Dis* 2017;8:353–61.

44 Curry N, Ravenscroft N. Countryside recreation provision in England : exploring a demand-led approach. *Land Use Policy* 2001;18:281–91.

45 Horner JS, Horner JW. Do doctors read forms? A one-year audit of medical certificates submitted to a crematorium. *J R Soc Med* 1998;91:371–6.

46 Department of Health. Health Protection Legislation (England) Guidance 2010. http://webarchive.nationalarchives.gov.uk/20130105053557/http://www.dh.gov.uk/prod_consum_dh/groups/dh_digitalassets/@dh/@en/@ps/documents/digitalasset/dh_114589.pdf (accessed 23 Oct 2018).

Figure Legends

Figure 1 - The annual incidence of Lyme disease in England and Wales (2013 -2016), and the

For peer review only - http://bmjopen.bmj.com/site/about/guidelines.xhtml

For peer review only

number of cases per month.

Figure 2 – Population demographics of laboratory-confirmed Lyme disease cases in England and Wales, 2013-2016. (Asterisks represent age bands with a significant difference between genders. Male = Blue, Female = Red.)

Figure 3 – The average incidence (cases per 100,000 per year) of laboratory-confirmed Lyme disease in England and Wales (2013-16)

(A = Public Health England region and Wales (n = 3,985), B = Patient postcode area (n = 2,321), C = Smoothed patient postcode area, D = LISA map of significant incidence clusters. Highest postcode areas and clusters are labelled accordingly; SO-Southampton, SP-Salisbury, BH-Bournemouth, RG-Reading, DT-Dorchester, GU-Guildford, TA-Taunton, TQ-Torquay, BN-Brighton, BA-Bath. Areas with no cases are labelled in red; DA-Dartford, EC-Eastern Central London, HU-Hull, and WC-Western Central London)

Figure 4 - Relationship between laboratory-confirmed Lyme disease case numbers (2013-2016) in England and the English Indices of Deprivation 2015.

License Statement

I, the Submitting Author has the right to grant and does grant on behalf of all authors of the Work (as defined in the below author licence), an exclusive licence and/or a non-exclusive licence for contributions from authors who are: i) UK Crown employees; ii) where BMJ has agreed a CC-BY licence shall apply, and/or iii) in accordance with the terms applicable for US Federal Government officers or employees acting as part of their official duties; on a worldwide, perpetual, irrevocable, royalty-free basis to BMJ Publishing Group Ltd ("BMJ") its

For peer review only - http://bmjopen.bmj.com/site/about/guidelines.xhtml

licensees and where the relevant Journal is co-owned by BMJ to the co-owners of the Journal, to publish the Work in BMJ Open and any other BMJ products and to exploit all rights, as set out in our licence.

The Submitting Author accepts and understands that any supply made under these terms is made by BMJ to the Submitting Author unless you are acting as an employee on behalf of your employer or a postgraduate student of an affiliated institution which is paying any applicable article publishing charge ("APC") for Open Access articles. Where the Submitting Author wishes to make the Work available on an Open Access basis (and intends to pay the relevant APC), the terms of reuse of such Open Access shall be governed by a Creative Commons licence – details of these licences and which Creative Commons licence will apply to this Work are set out in our licence referred to above.

For peer review only

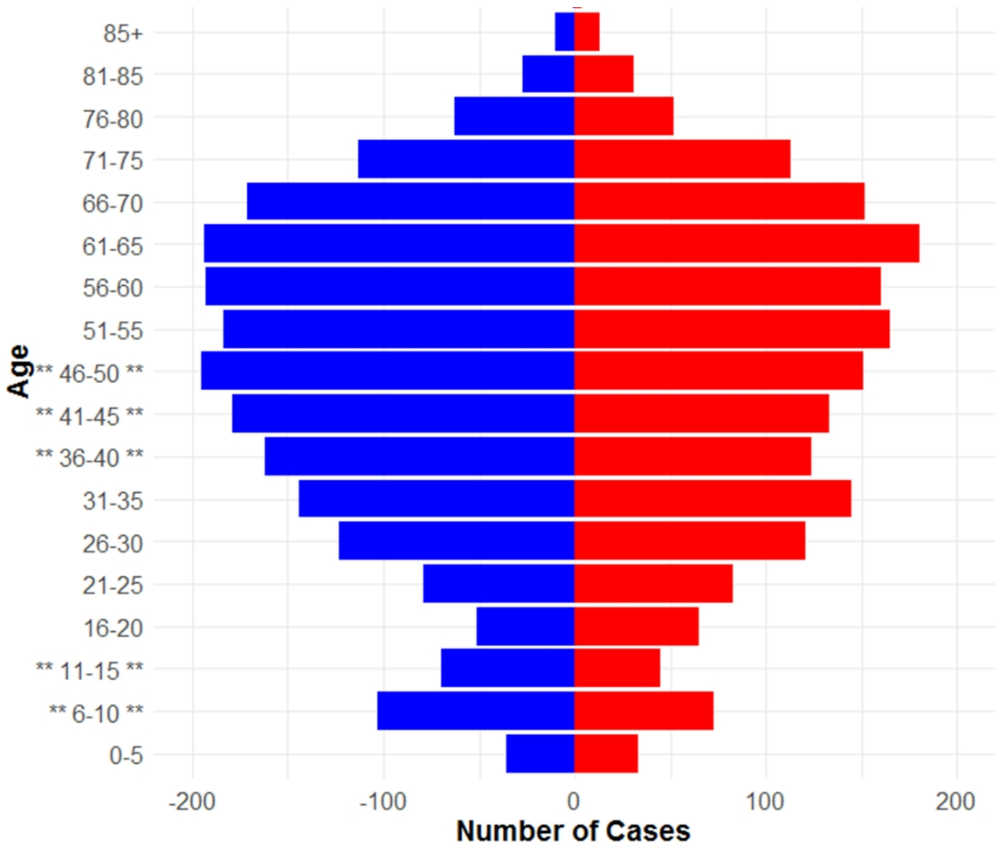

Figure 1 - The annual incidence of Lyme disease in England and Wales (2013 -2016), and the number of cases per month.

1116x939mm (96 x 96 DPI)

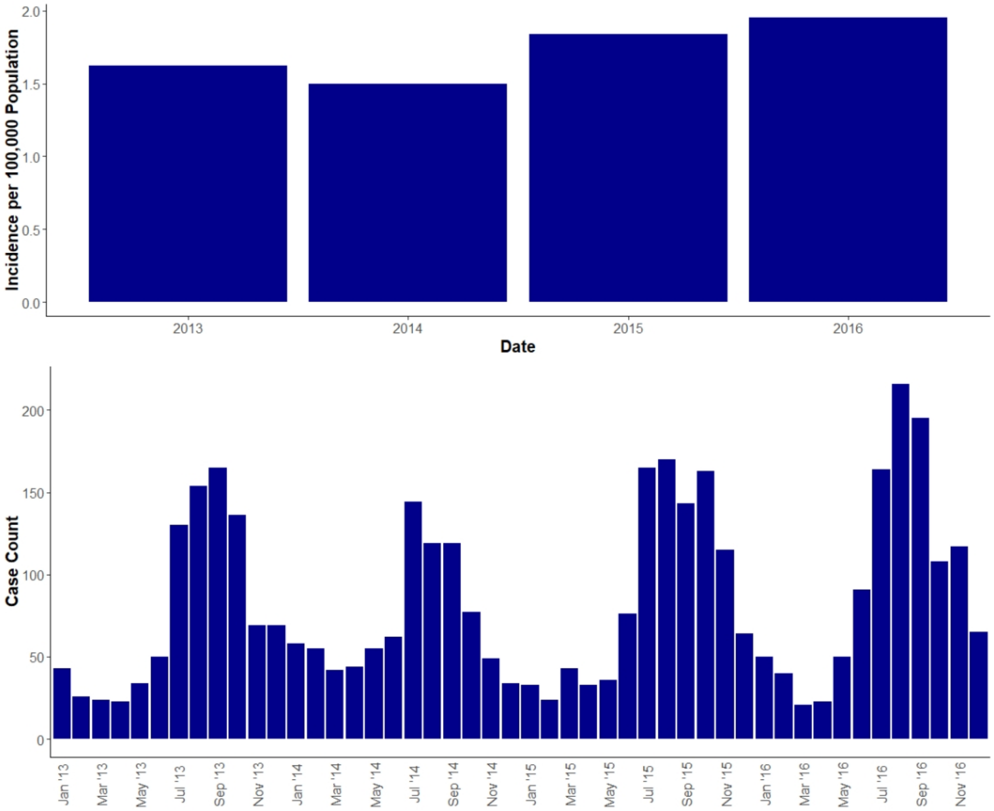

Figure 2 – Population demographics of laboratory-confirmed Lyme disease cases in England and Wales, 2013-2016. (Asterisks represent age bands with a significant difference between genders. Male = Blue, Female = Red.)

319x260mm (96 x 96 DPI)

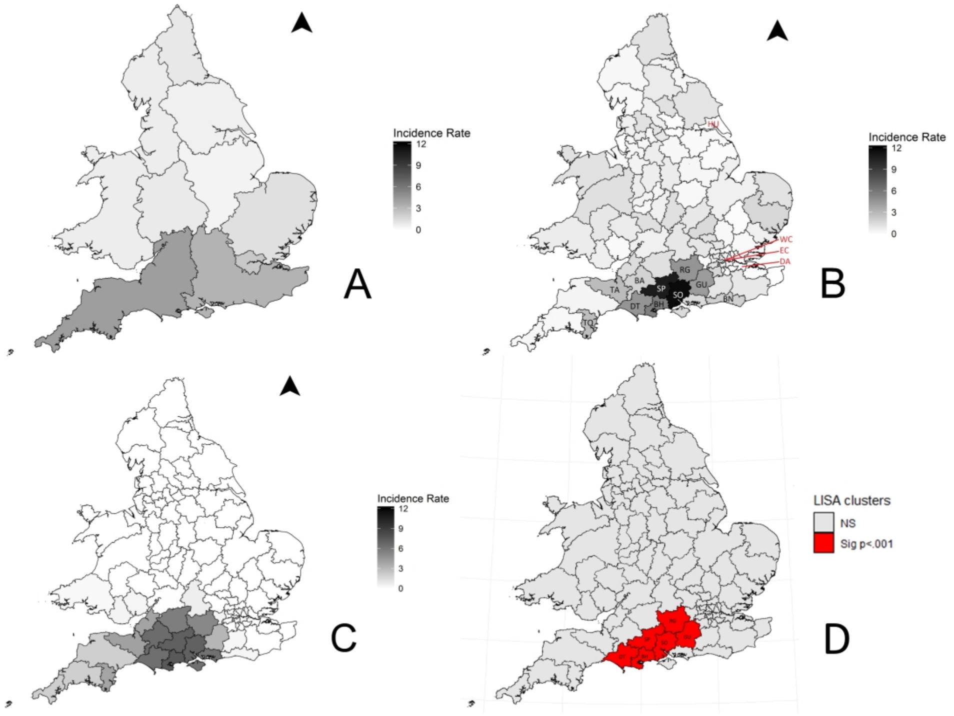

Figure 3 – The average incidence (cases per 100,000 per year) of laboratory-confirmed Lyme disease in England and Wales (2013-16) (A = Public Health England region and Wales (n = 3,985), B = Patient postcode area (n = 2,321), C = Smoothed patient postcode area, D = LISA map of significant incidence clusters. Highest postcode areas and clusters are labelled accordingly; SO-Southampton, SP-Salisbury, BH-Bournemouth, RG-Reading, DT-Dorchester, GU-Guildford, TA-Taunton, TQ-Torquay, BN-Brighton, BA-Bath. Areas with no cases are labelled in red; DA-Dartford, EC-Eastern Central London, HU-Hull, and WC-Western Central London)

527x381mm (150 x 150 DPI)

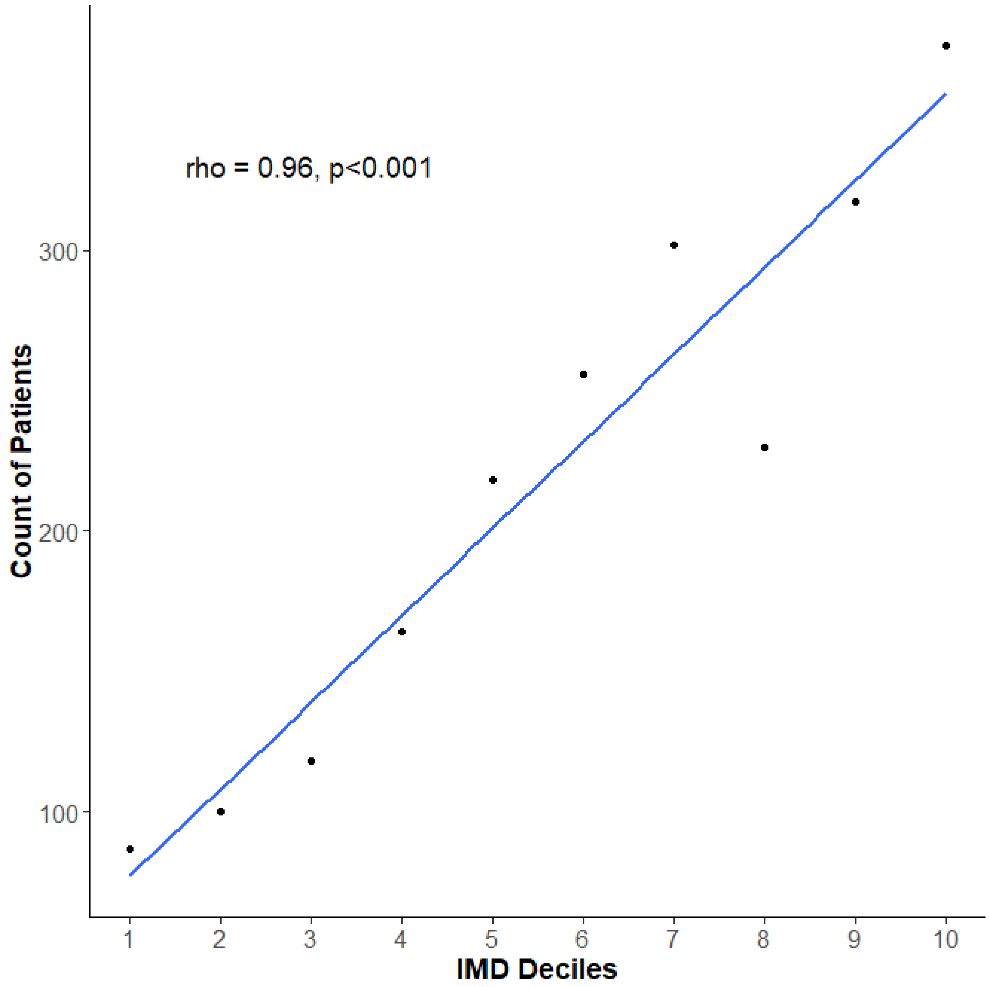

Figure 4 - Relationship between laboratory-confirmed Lyme disease case numbers (2013-2016) in England and the English Indices of Deprivation 2015.

178x178mm (96 x 96 DPI)

Supplementary Material 1

The English Indices of Deprivation (EID) ranks 32,844 geographies (Lower super output areas) containing between 1000 and 3000 population and groups these in to deciles where 1 represents the areas with the highest levels of deprivation and 10 the lowest. The Welsh Index of Multiple Deprivation (WIMD) is ranked in a similar manner but is then grouped into the following categories; the 10% with the greatest deprivation, moving up through decreasing levels of deprivation in intervals from 10-20%, 20-30%, 30-50%, and the 50% least deprived areas. The EID and WIMD categorise each geographical area with a variety of deprivation domain scores to build a summary index figure (Supplementary Table 1).

For peer review only

Supplementary Table 1. Summary of the English Indices of Deprivation 2015[33] and Welsh Index of Multiple Deprivation[34] domains, and their weighting to calculate an Index of Multiple Deprivation

**English Indices of Deprivation (EID) Domain**	**Description**	**Weighting for construction of Index of Multiple Deprivation (IMD)**
Income Deprivation Domain	Proportion of population experiencing deprivation due to low income	22.5%
Employment Deprivation Domain	Proportion of working age population excluded from the labor market	22.5%
Education, Skills and Training Deprivation Domain	Measures the lack of attainment and skills in the local population	13.5%
Health Deprivation and Disability Domain	Measures the risk of premature death and the impairment of quality of life through poor physical or mental health	13.5%
Crime Domain	Measures the risk of personal and material victimisation at local level	9.3%
Barriers to Housing and Services Domain	Measures the physical and financial accessibility of housing and local services (schools, supermarkets, primary care and post offices)	9.3%
Living Environment Deprivation Domain	Measures the quality of the local environment (housing, air quality and road traffic accidents)	9.3%
**Index of Multiple Deprivation (IMD)**	**Overall measure of deprivation constructed by the weighted sum of the above domains**	
**Welsh Index of Multiple Deprivation (WIMD) Domain**	**Description**	**Weighting for construction of Welsh Index of Multiple Deprivation (WIMD)**
Income Domain	Proportion of population experiencing deprivation due to low income	23.5%
Employment Domain	Proportion of working age population excluded from the labor market	23.5%
Health Domain	Measures the lack of good health	14.0%
Education Domain	Measures the extent of deprivation relating to education, training and skills	14.0%
Access to Services Domain	Measures deprivation due to a house holds inability to access services considered necessary for day to day living.	10.0%
Community Safety Domain	Measures deprivation relating to living in a safe community	5.0%
Physical Environment Domain	Measures factors in the local area that may impact on wellbeing or quality of life	5.0%
Housing Domain	Measures deprivation through lack of adequate housing	5.0%
**Welsh Index of Multiple Deprivation (WIMD)**	**Overall measure of deprivation constructed by the weighted sum of the above domains**	

For peer review only

Supplementary Material 2

Supplementary Figure - Relationship between laboratory-confirmed Lyme disease case numbers (2013-2016) in England and the component measures of the English Indices of Deprivation 2015.

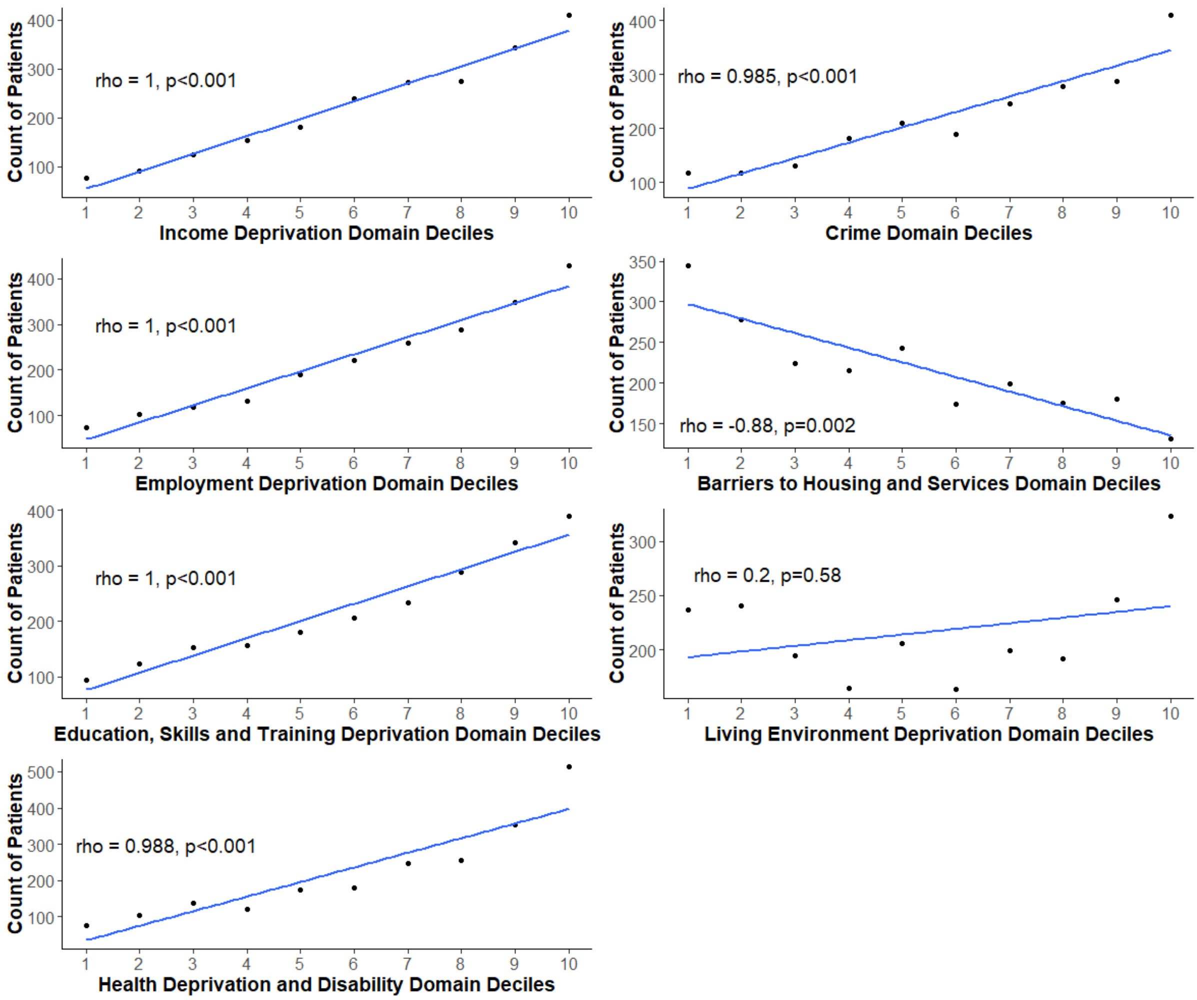

STROBE 2007 (v4) checklist of items to be included in reports of observational studies in epidemiology*
Checklist for cohort, case-control, and cross-sectional studies (combined)

Section/Topic	Item #	Recommendation	Reported on page #
**Title and abstract**	1	(*a*) Indicate the study's design with a commonly used term in the title or the abstract	2
		(*b*) Provide in the abstract an informative and balanced summary of what was done and what was found	2
**Introduction**			
Background/rationale	2	Explain the scientific background and rationale for the investigation being reported	4-6
Objectives	3	State specific objectives, including any pre-specified hypotheses	6
**Methods**			
Study design	4	Present key elements of study design early in the paper	6
Setting	5	Describe the setting, locations, and relevant dates, including periods of recruitment, exposure, follow-up, and data collection	6
Participants	6	(*a*) *Cohort study*—Give the eligibility criteria, and the sources and methods of selection of participants. Describe methods of follow-up<br>*Case-control study*—Give the eligibility criteria, and the sources and methods of case ascertainment and control selection. Give the rationale for the choice of cases and controls<br>*Cross-sectional study*—Give the eligibility criteria, and the sources and methods of selection of participants	6
		(*b*) *Cohort study*—For matched studies, give matching criteria and number of exposed and unexposed<br>*Case-control study*—For matched studies, give matching criteria and the number of controls per case	N/A
Variables	7	Clearly define all outcomes, exposures, predictors, potential confounders, and effect modifiers. Give diagnostic criteria, if applicable	6-8
Data sources/ measurement	8*	For each variable of interest, give sources of data and details of methods of assessment (measurement). Describe comparability of assessment methods if there is more than one group	6-8
Bias	9	Describe any efforts to address potential sources of bias	N/A
Study size	10	Explain how the study size was arrived at	6
Quantitative variables	11	Explain how quantitative variables were handled in the analyses. If applicable, describe which groupings were chosen and why	6-8
Statistical methods	12	(*a*) Describe all statistical methods, including those used to control for confounding	6-8
		(*b*) Describe any methods used to examine subgroups and interactions	N/A
		(*c*) Explain how missing data were addressed	N/A
		(*d*) *Cohort study*—If applicable, explain how loss to follow-up was addressed<br>*Case-control study*—If applicable, explain how matching of cases and controls was addressed	N/A

For peer review only

		Cross-sectional study—If applicable, describe analytical methods taking account of sampling strategy	
		(*e*) Describe any sensitivity analyses	N/A
**Results**			
Participants	13*	(a) Report numbers of individuals at each stage of study—eg numbers potentially eligible, examined for eligibility, confirmed eligible, included in the study, completing follow-up, and analysed	8
		(b) Give reasons for non-participation at each stage	N/A
		(c) Consider use of a flow diagram	N/A
Descriptive data	14*	(a) Give characteristics of study participants (eg demographic, clinical, social) and information on exposures and potential confounders	8-10
		(b) Indicate number of participants with missing data for each variable of interest	8-10
		(c) *Cohort study*—Summarise follow-up time (eg, average and total amount)	N/A
Outcome data	15*	*Cohort study*—Report numbers of outcome events or summary measures over time	N/A
		Case-control study—Report numbers in each exposure category, or summary measures of exposure	N/A
		Cross-sectional study—Report numbers of outcome events or summary measures	8-10
Main results	16	(*a*) Give unadjusted estimates and, if applicable, confounder-adjusted estimates and their precision (eg, 95% confidence interval). Make clear which confounders were adjusted for and why they were included	8-10
		(*b*) Report category boundaries when continuous variables were categorized	8-10
		(*c*) If relevant, consider translating estimates of relative risk into absolute risk for a meaningful time period	N/A
Other analyses	17	Report other analyses done—eg analyses of subgroups and interactions, and sensitivity analyses	8-10
**Discussion**			
Key results	18	Summarise key results with reference to study objectives	10-11
Limitations	19	Discuss limitations of the study, taking into account sources of potential bias or imprecision. Discuss both direction and magnitude of any potential bias	14-15
Interpretation	20	Give a cautious overall interpretation of results considering objectives, limitations, multiplicity of analyses, results from similar studies, and other relevant evidence	15-16
Generalisability	21	Discuss the generalisability (external validity) of the study results	15-16
**Other information**			
Funding	22	Give the source of funding and the role of the funders for the present study and, if applicable, for the original study on which the present article is based	16

*Give information separately for cases and controls in case-control studies and, if applicable, for exposed and unexposed groups in cohort and cross-sectional studies.

Note: An Explanation and Elaboration article discusses each checklist item and gives methodological background and published examples of transparent reporting. The STROBE checklist is best used in conjunction with this article (freely available on the Web sites of PLoS Medicine at http://www.plosmedicine.org/, Annals of Internal Medicine at http://www.annals.org/, and Epidemiology at http://www.epidem.com/). Information on the STROBE Initiative is available at www.strobe-statement.org.

For peer review only
